# Characterization of the Mycobacterial Acyl-CoA Carboxylase Holo Complexes Reveals Their Functional Expansion into Amino Acid Catabolism

**DOI:** 10.1371/journal.ppat.1004623

**Published:** 2015-02-19

**Authors:** Matthias T. Ehebauer, Michael Zimmermann, Arjen J. Jakobi, Elke E. Noens, Daniel Laubitz, Bogdan Cichocki, Hedia Marrakchi, Marie-Antoinette Lanéelle, Mamadou Daffé, Carsten Sachse, Andrzej Dziembowski, Uwe Sauer, Matthias Wilmanns

**Affiliations:** 1 European Molecular Biology Laboratory, Hamburg Unit, Hamburg, Germany; 2 Institute of Molecular Systems Biology, ETH Zurich, Zurich, Switzerland; 3 European Molecular Biology Laboratory, Structural Biology and Computational Biology Programme, Heidelberg, Germany; 4 Institute of Biochemistry and Biophysics, Polish Academy of Sciences, Warsaw, Poland; 5 Department of Genetics & Biotechnology, Warsaw University, Warsaw, Poland; 6 Centre National de la Recherche Scientifique, Institut de Pharmacologie et de Biologie Structurale, Tuberculosis & Infection Biology Department, Toulouse, France; Université Paul Sabatier, Toulouse, France; 7 Center for Structural Systems Biology, Hamburg, Germany; Weill Medical College of Cornell University, UNITED STATES

## Abstract

Biotin-mediated carboxylation of short-chain fatty acid coenzyme A esters is a key step in lipid biosynthesis that is carried out by multienzyme complexes to extend fatty acids by one methylene group. Pathogenic mycobacteria have an unusually high redundancy of carboxyltransferase genes and biotin carboxylase genes, creating multiple combinations of protein/protein complexes of unknown overall composition and functional readout. By combining pull-down assays with mass spectrometry, we identified nine binary protein/protein interactions and four validated holo acyl-coenzyme A carboxylase complexes. We investigated one of these - the AccD1-AccA1 complex from *Mycobacterium tuberculosis* with hitherto unknown physiological function. Using genetics, metabolomics and biochemistry we found that this complex is involved in branched amino-acid catabolism with methylcrotonyl coenzyme A as the substrate. We then determined its overall architecture by electron microscopy and found it to be a four-layered dodecameric arrangement that matches the overall dimensions of a distantly related methylcrotonyl coenzyme A holo complex. Our data argue in favor of distinct structural requirements for biotin-mediated γ-carboxylation of α−β unsaturated acid esters and will advance the categorization of acyl-coenzyme A carboxylase complexes. Knowledge about the underlying structural/functional relationships will be crucial to make the target category amenable for future biomedical applications.

## Author Summary

Tuberculosis is deadly human disease caused by infection with the bacterium *Mycobacterium tuberculosis*. This pathogen has a complex metabolism with many genes required for the synthesis of components of its unique cell envelope. We have investigated a family of closely related genes coding for different acyl CoA carboxylase enzyme complexes with previously unexplained genetic redundancy that have been thought to have an involvement in the synthesis of these cell envelope components. We identified five functional multienzyme complexes. Of the two complexes with hitherto unknown function we chose to investigate, one specifically and to our surprise it is required for the degradation of the amino acid leucine. To our knowledge this is the first demonstration that mycobacteria have a specific pathway for leucine degradation and thus broaden the functional diversity associated with acyl CoA carboxylase coding genes.

## Introduction

Biotin-dependent acyl-CoA carboxylases (YCCs) are ubiquitous multifunctional enzymes that are found in all kingdoms of life. They have key roles in the metabolism of fatty acids, amino acids, and carbohydrates and have various, more specialized functions in microorganisms [1,2]. YCC substrates are generally coenzyme A [3] esters, in which the site for carboxylation is either the α-carbon position of saturated acid esters, such as acetyl-CoA and propionyl-CoA, or the γ-carbon position of α-β unsaturated acid esters. The reaction schemes of the YCCs all have two sequential steps in common: first, the biotin post-translationally attached to a biotin carboxyl carrier protein (BCCP) is carboxylated in an ATP-dependent manner by a biotin carboxylase (BC); second, carboxybiotin attached to the BCCP is transferred to the active site of the carboxyltransferase (CT), where the transfer of the carboxyl group from biotin to the acyl-CoA substrate takes place to extend the acyl-CoA by one carboxyl group. Depending on the organism, there are large variations in how these enzymatic activities are arranged, from single multifunctional, multidomain sequences to multisubunit oligomeric assemblies that have separate subunit activities.

Human YCCs have already been established as primary drug targets against diseases such as cancer and type II diabetes, and plastid YCCs have been targeted by commercialized herbicides for several decades [2]. The BC enzymatic activity of bacterial YCCs has also been successfully used for the identification of potent inhibitors against Gram-negative bacteria [4]. However, despite a significant number of attempts, there has been no breakthrough in using YCCs from mycobacteria as drug targets [5]. One of the reasons for this lack of progress is that there is no structural and functional repository of all mycobacterial YCC complexes, which would allow the development of specific approaches against individual members.

In actinomycetes, YCCs are generally organized into oligomeric assemblies composed of two different subunits: the α-subunit comprises the BC activity and the β-subunit is responsible for the CT activity. In *Mycobacterium tuberculosis* and related mycobacteria, there is an unusual redundancy in both genes, with three different YCC α-subunits and six different β-subunits being encoded, named AccA1 to AccA3 and AccD1 to AccD6, respectively. Whereas all three α-subunit sequences share more than 40% sequence identity in *M*. *tuberculosis* and *M*. *smegmatis*, the β-subunit CT sequences that determine the acyl-CoA substrate specificity are more divergent and can be categorized into three groups (S1 Table): the first comprises AccD1 and AccD2; the second includes AccD4, AccD5 and AccD6; and the third is AccD3, which shares less than 20% sequence identity with other β-subunit members.

The principle substrates for two of the six *M*. *tuberculosis* YCC β-subunits, AccD6 and AccD5, have been identified as acetyl-CoA and propionyl-CoA, respectively [6,7,8]. Acetyl-CoA YCC carboxylation generates malonyl-CoA, which is a main building block in fatty-acid biosynthesis. Propionyl-CoA carboxylation leads to methylmalonyl-CoA, which is essential for the synthesis of the methyl-branched lipids of the outer mycobacterial cell wall and capsule, and is an intermediate in the methylmalonyl pathway to catabolize propionyl-CoA [9,10,11]. The mycobacterial AccD4-containing YCC complex carboxylates long-chain acyl-CoA, which is required for the biosynthesis of unusual very long-chain fatty acids such as mycolic acid [12,13]. Based on these findings, YCC redundancy in mycobacteria was generally thought of being related to the complex requirements of lipid biosynthesis pathways [5,14].

Previous molecular YCC interaction studies have been limited to the AccA3 BC subunit, which revealed oligomeric complexes with oligomeric complexes with integer multiples of a 1:1 α:β subunit stochiometry with CT subunits AccD4, AccD5, and AccD6 [6,15,16]. A systematic analysis of the YCC interactions in mycobacteria is still required to establish an integrative view on the overall functional portfolio of YCC complexes. Here, we have addressed this gap along with the resulting mechanistic questions by combining genetic, proteomic, lipidomic, metabolomic, biochemical and structural approaches.

Our interaction screen revealed nine binary protein/protein interactions that generate at least four different mycobacterial YCC holo complexes. From these identified assemblies, the AccD1 (Rv2502c)-AccA1 (Rv2501c) YCC complex was selected for functional and structural studies. We demonstrate that this complex encodes a 3-methylcrotonyl-CoA carboxylase (MCC) involved in leucine catabolism. Electron micrographs of the AccD1-AccA1 holo complex reveal the general architecture of an MCC. In summary, our data show that mycobacterial YCC redundancy adds unexpected functional diversity in both α-carbon and γ-carbon acyl-CoA carboxylation biochemistry, reflected by distinct YCC structural arrangements.

## Results

### Protein/protein interaction map of YCC complexes

To rationalize our selection of mycobacterial YCC complexes for mechanistic studies, we first identified a complete set of protein/protein interactions of mycobacterial YCC genes. We used all nine known mycobacterial YCC BT (α-subunit) and CT (β-subunit) genes to perform pull-down experiments in *M*. *smegmatis*, which has both comparable YCC gene organization and high sequence identity to their respective loci in *M*. *tuberculosis* (S1 Table). We also included the YCC gene coding for a small ε-subunit that has been shown to be involved in the formation of some YCC holo complexes and to act as a potential regulator of activity [5,6,13,16]. All ten *M*. *smegmatis* genes were tagged with C-terminal enhanced Green Fluorescent Protein (eGFP). The C-terminus of each α-subunit was selected for tagging, as it is located in the BCCP domain, which is next to the flexible linker allowing the BCCP to move between the active sites of the complex. Any interference of the eGFP tag with the BC/CT protein/protein interactions is hence expected to be minimal. The C-terminus of each β-subunit was tagged, as the N-terminus is located at the BC-CT interface [17]. Interacting protein partners of enriched eGFP-fusion proteins were identified by liquid chromatography-coupled tandem mass spectrometry (LC-MS/MS) (S2 Table). As a control, we found strong self-assembly of all gene products tested, which is in line with various data on the involvement of both BC and CT subunit oligomerization in YCC holo complex formation [5].

**Fig 1:**
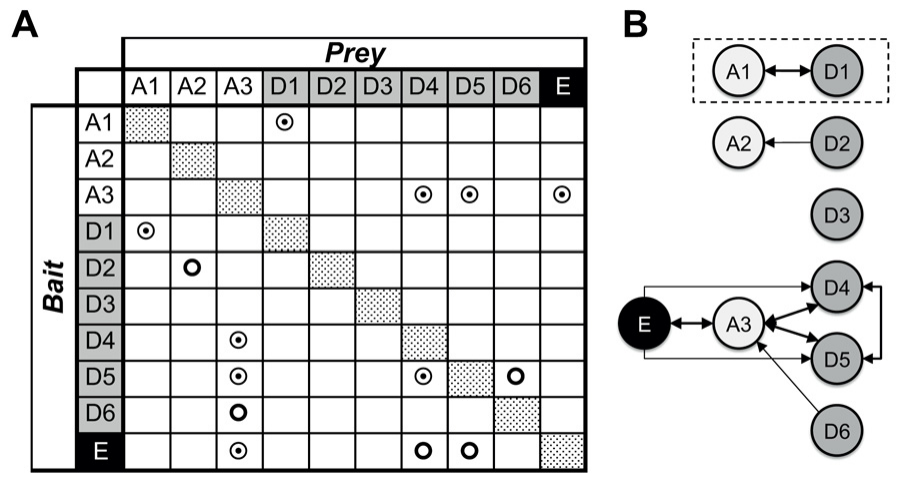
Mycobacterial YCC protein/protein interactions. (*A*) Binary, hetero-dimeric protein/protein interactions are indicated by double circles when found reciprocally, by using reverse bait/prey protein pairs, and by simple circles when identified only in one of the two possible combinations. Homo-oligomeric assemblies were found for all proteins tested (dotted patterns). Mycobacterial YCC CT subunits AccD1 to AccD6 are denoted “D1” to “D6” (grey), BT subunits AccA1 to AccA3 are denoted “A1” to “A3” (no background), and the AccE ε-subunit is labeled “E” (black). (*B*) Identification of YCC holo complexes. Arrows point to the protein component used as prey; protein/protein assemblies, for which binary interactions were identified by the reverse use of bait/prey are indicated by thick double arrows. For further details, see S2 Table.

In total, we found nine binary protein/protein interactions, of which five were confirmed by the reverse experiment that swapped bait and prey functions of interacting protein partners (Fig. 1, S2 Table). Such reciprocal interactions between a BC α-subunit and a CT β-subunit were identified for the protein pairs AccD1-AccA1, AccD4-AccA3, AccD5-AccA3. In addition, a reciprocal interaction between the AccA3 BC α-subunit and the small AccE ε-subunit was found. When using AccE as bait, interactions could also be identified with AccD4 and AccD5, confirming that the ε-subunit gene is involved in the formation of the AccD4-AccA3 and AccD5-AccA3 holo complexes [6,12]. Reciprocal interactions for the CT pair AccD4-AccD5 was also observed, confirming previous interaction data centered on the mycobacterial AccD4 subunit [12]. These findings are suggestive of a mixed ε-subunit-mediated AccD4/AccD5/AccA3 holo complex that unfortunately has not yet been isolated at sufficient purity for further functional and structural studies [6].

Non-reciprocal interactions were found for the pairs AccD2-AccA2 and AccD6-AccA3, using AccA2 and AccD6 as bait, respectively. Despite a sequence identity of more than 50% between AccD6 and AccD5, no direct interaction between AccD6 and AccE could be detected (S1 Table). This suggests that the involvement of the AccE ε-subunit is limited to the formation of the AccD4-AccA3 and AccD5-AccA3 holo complexes and points to a different structural and, hence, most likely functional role for AccD6. AccD3 did not interact with any protein partner used in this analysis, raising doubts as to whether this protein indeed functions as a CT subunit in a YCC holo complex [18]. A separate functional role for AccD3 may also be inferred from the low sequence similarity of 22% or less with the other CT subunits, which share 28–52% similarity among each other (S1 Table).

Taking these pull-down and sequence comparison data together, our findings indicate that the YCCs of mycobacteria consist of at least two clusters of YCC holo complexes: two highly related complexes that do not require the ε-subunit (AccD1-AccA1, AccD2-AccA2) and another two complexes (AccD4-AccA3, AccD5-Acc3) that are consistently associated with the ε-subunit AccE. The involvement of the two remaining proteins (AccD3, AccD6) in YCC holo complexes is less clear, questioning their precise role in established YCC functions that require the presence of a BT carboxybiotin donor. By making use of the so established mycobacterial YCC interaction map, we subsequently selected the AccD1-AccA1 YCC holo complex for mechanistic studies as the formation of this complex had been verified reversibly and its functional role was unknown.

### AccA1 and AccD1 form a high molecular weight protein/protein complex *in vitro*

**Fig 2:**
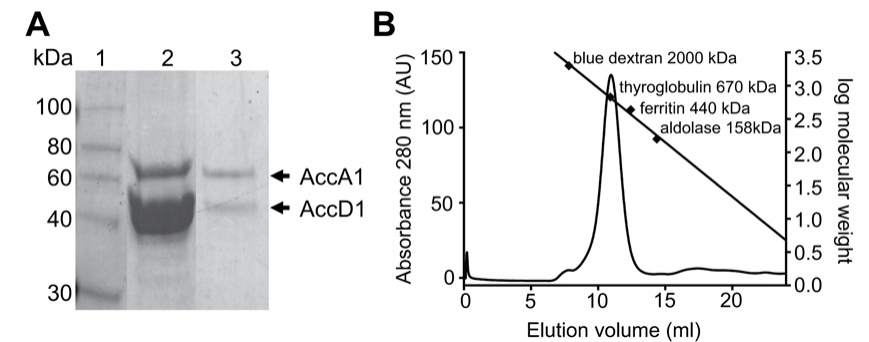
AccD1-AccA1 holo complex formation. (A) SDS-PAGE showing the Ni-affinity eluate (lane 2), and after gel filtration (panel B) in lane 3; molecular markers are shown in Lane 1. (B) Size-exclusion chromatogram of purified AccD1-AccA1. The elution volume and molecular mass of the molecular weight calibration standards are indicated. The equation describing the linear regression line is f(x) = -0.16x + 4.6 and has a R2 = 0.99.

To generate an efficient AccD1-AccA1 holo complex purification protocol we took advantage of the operon organization of the *accD1* and *accA1* genes, which are part of the *citE-scoA* operon (Rv2498c to Rv2504c in *M*. *tuberculosis;* MS4713 to MS4716 in *M*. *smegmatis*, respectively) (S1 Fig.). Putative ribosome-binding sites are only found in *accD1* upstream of *accA1* and hence we cloned the two *M*. *tuberculosis* genes as a single bicistronic sequence into an expression plasmid for heterologous co-expression in *M*. *smegmatis* to mimic their natural operon organization. A hexa-histidine tagged AccD1 version was used for the purification of the AccD1-AccA1 complex. When the cleared cell lysate was subjected to a Ni-affinity matrix, both AccD1 and AccA1 could be extracted demonstrating a direct interaction between the two subunits, and they had an apparent molecular mass of approximately 45 kDa and 65 kDa, respectively (Fig. 2A). The identity of the two proteins was verified by peptide mass fingerprinting.

Further evidence of a direct protein/protein interaction between AccD1 and AccA1 was obtained by loading the affinity-purified proteins onto a size-exclusion column. Both proteins co-eluted as a single symmetric peak, indicating that the protein complex has a defined, homogenous association state (Fig. 2B). According to calibration mass standards, the molecular mass of the protein complex is around 700 kDa, suggesting that there are either five or six AccD1-AccA1 heterodimers corresponding to a calculated molecular mass of 637 kDa and 764 kDa, respectively. The same co-purification protocol was applied to confirm the interaction and demonstrate complex formation of the AccD2-AccA2 complex, which we used as a control for subsequent functional studies (S2 Fig.).

### AccD1-AccA1 is involved in the leucine degradation pathway and carboxylates 3-methylcrotonyl-CoA

To identify the function of the AccD1-AccA1 complex in mycobacteria, we generated knockout strains of *M*. *smegmatis accD1*-*accA1* and their close paralogues *accD2-accA2*. Growth of both strains was comparable to wild type (wt) *M*. *smegmatis* in 7H9 medium supplemented with glycerol indicating that both complexes are dispensable under standard *in vitro* growth conditions (S3 Fig.).

We first tested a possible role for AccD1-AccA1 in lipid biosynthesis. When comparing the lipid composition in wt *M*. *smegmatis*, Δ*accD1-*Δ*accA1* and Δ*accD2-*Δ*accA2* strains we found no significant differences in the composition and concentration of either fatty acid methyl esters (FAMEs) analyzed by thin-layer chromatography (TLC) and gas chromatography—mass spectrometry (GC-MS) or mycolic acids analyzed by TLC and MALDI-TOF mass spectrometry (S3 Table).

We then searched for potential metabolic intermediates in *de novo* fatty acid and mycolic acid synthesis by [^14^C]acetate tracing of fatty acids and mycolic acids during the growth of wt *M*. *smegmatis* and the two mutant strains. TLC analysis of newly synthesized compounds indicated that fatty acids and all species of mycolic acids characteristic for *M*. *smegmatis* are present in all three strains. No additional radioactive lipid intermediates, such as meromycolic acid and alkyl-malonic acid, could be observed (S4 Fig.). The absence of alkyl-malonic acids was confirmed by GC-MS analysis of the cold total FAME pool [12]. Based on these findings from volatile and non-volatile lipid analysis, we concluded that neither AccD1-AccA1 nor AccD2-AccA2 complexes have a direct role in lipid metabolism.

**Fig 3:**
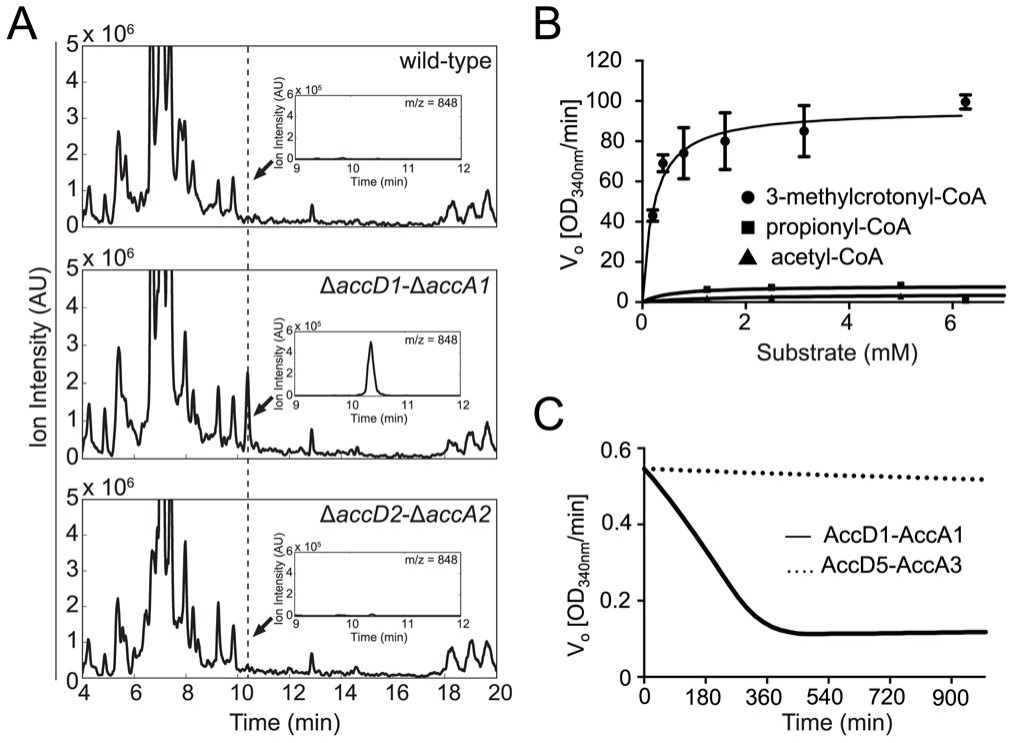
Identification and biochemical characterization of the AccD1-AccA1 substrate 3-methylcrotonyl-CoA. (A) Untargeted CoA-profiles of YCC knockout strains. Mass spectra of Co-A profiles of M. smegmatis wt and knockout strains. Insets show spectra at dashed line. The peak evident in the ΔaccD1-ΔaccA1 spectrum is that of 3-methylcrotonyl-CoA. (B) Steady-state kinetics of AccD1-AccA1 incubated with 3-methylcrotonyl-CoA, propionyl-CoA and acetyl-CoA. (C) Time course of the consumption of 3-methylcrotonyl-CoA by purified M. tuberculosis AccD1-AccA1 and AccD5-AccA3.

To subsequently test for potential alternative metabolic functions of the AccD1-AccA1 complex, we performed metabolic profiling specific to CoA thioesters in wt *M*. *smegmatis* and the Δ*accD1-*Δ*accA1* and Δ*accD2-*Δ*accA2* deletion strains by liquid chromatography—tandem mass spectrometry (LC-MS/MS) [19]. In these CoA-specific metabolite profiles we identified a single additional peak in the Δ*accD1-*Δ*accA1* extracts relative to the other two strains (Fig. 3A). Based on its mass and retention time, this peak was assigned to 3-methylcrotonyl-CoA (3-methylbut-2-enoyl-CoA) suggesting that the AccD1-AccA1 holo complex functions as a methylcrotonyl-CoA carboxylase [20].

To confirm this finding biochemically, we used the recombinant *M*. *tuberculosis* AccD1-AccA1 complex and 3-methylcrotonyl-CoA as the substrate in an *in vitro* enzymatic activity assay. As the enzyme assay measures carboxylation activity indirectly, we first determined the formation of the reaction product 3-methylglutaconyl-CoA demonstrating that the assay is useful in determining kinetic parameters (S5 Fig.). The K_M_ of the AccD1-AccA1 complex for 3-methylcrotonyl-CoA turnover was determined as 0.22 ± 0.05 mM, and the k_cat_/K_m_ as 303 ± 15 mM^-1^ s^-1^ (Fig. 3B). Only residual enzyme activity could be measured for propionyl-CoA and acetyl-CoA demonstrating the specificity of the enzyme for γ-carbon carboxylation. As an additional control, we also tested purified AccD5-AccA3 complex for 3-methylcrotonyl-CoA carboxylation but did not find any measurable activity, demonstrating that the two enzyme systems do not overlap (Fig. 3C). Our biochemical data are thus in agreement with the metabolomics findings and the growth characterization and therefore support a role for the AccD1-AccA1 complex as an MCC.

**Fig 4:**
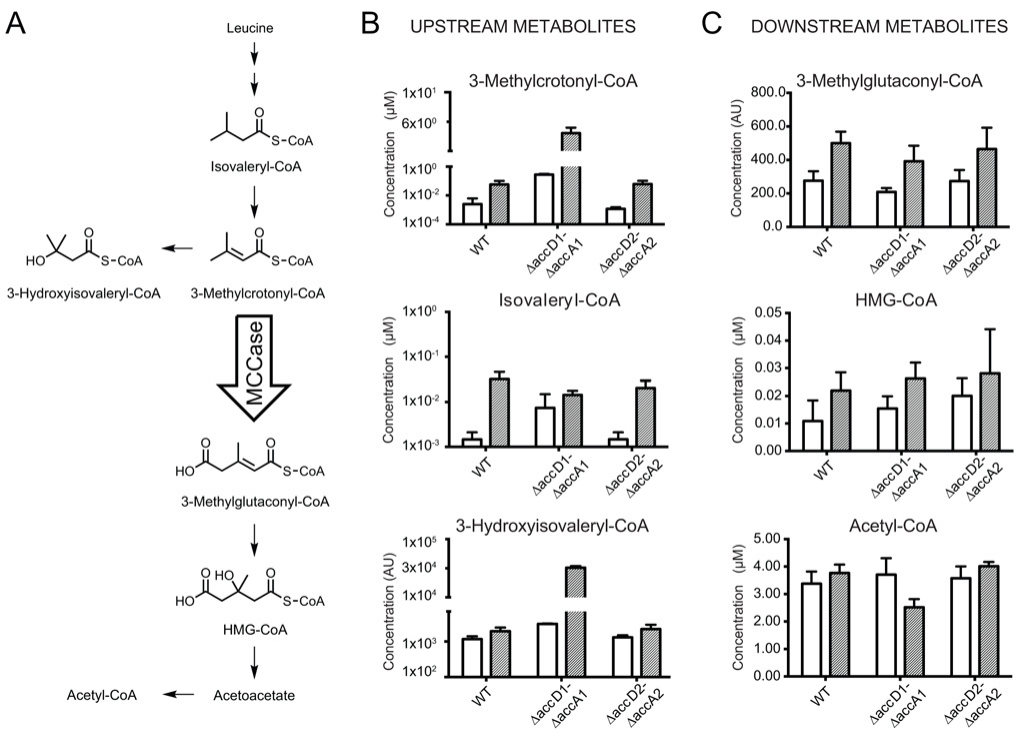
Metabolite analysis upstream and downstream of 3-methylcrotonyl-CoA. (A) The leucine degradation pathway in eubacteria, modified from pathway ko00280 of the KEGG database (www.kegg.jp/kegg-bin/show_pathway?map00280). (B) Metabolites upstream of the putative MCC activity. The metabolite 3-methylcrotonyl-CoA accumulates during leucine degradation in ΔaccD1-ΔaccA1 M. smegmatis. However, this does not lead to backlog accumulation of isovaleryl-CoA upstream of MCC catalysis during leucine degradation. An increased pool of 3-methylcrotonyl-CoA upon MCC blockage also triggers accumulates of hydroxyisovaleryl-CoA. (C) Metabolites downstream of the AccD1-AccA1 MCC activity: Both methylglutaconyl-CoA and HMG-CoA show generally higher levels during leucine-degrading conditions. The amounts are comparable between wt and ΔaccD1-ΔaccA1 strains, as one would expect for metabolites downstream of a blocked enzymatic function. For compounds that are commercially available, chemical standard metabolite concentrations in the metabolite extracts are given in μM. Otherwise, relative concentrations are indicated and the compounds are annotated based on their mass. Average concentrations and standard deviations were calculated from four independent bacterial cultures of each strain.

To further corroborate these findings, we quantified the metabolites of the canonical leucine-degradation pathway, in which 3-methylcrotonyl-CoA is one of the substrates (Fig. 4A, S6 Fig.). We investigated wt *M*. *smegmatis* and the two knockout strains under glycerol and leucine catabolizing growth conditions (Fig. 4B—C). When transferred to culture media containing leucine as the carbon source, 3-methylcrotonyl-CoA accumulated in the Δ*accD1-*Δ*accA1* strain to levels approximately 100 times above those in wt and the Δ*accD2-*Δ*accA2* strain (Fig. 4B, grey bars). The blockage of the metabolic pathway at the level of the MCC activity did not lead to the accumulation of isovaleryl-CoA upstream of 3-methylcrotonyl-CoA, as there is little difference between its levels in the wt and Δ*accD1-*Δ*accA1* strains (Fig. 4B). This observation could be explained by alternative reactions consuming methylcrotonyl-CoA to form 3-hydroxyisovaleryl-CoA, the levels of which also increase in the Δ*accD1-*Δ*accA1* strain (Fig. 4B). The accumulation of metabolites directly upstream in the pathway of an impaired enzymatic activity is a common phenomenon and, in general, is a valid parameter to localize enzymatic function [21][21]. Consistently, the levels of 3-methylglutaconyl-CoA and 3-hydroxy-3-methylglutaryl (HMG)-CoA, localized downstream of the MCC, were similar in all strains (Fig. 4C). Acetyl-CoA is the final product of leucine degradation and is involved in many different metabolic pathways. Its level in the Δ*accD1-*Δ*accA1* strain cultivated in leucine-containing media is lower than that of the wt strain, which could be caused by impaired viability (Fig. 4C, grey bars). These metabolomics data thus further demonstrate that AccD1-AccA1 has MCC activity and is involved in leucine catabolism.

**Fig 5:**
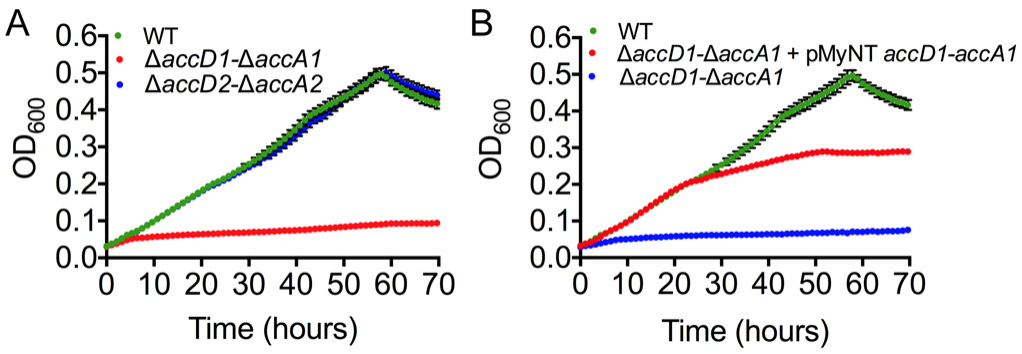
Growth phenotype characterization and complementation of mycobacterial accD1 and accA1 genes. (A) Growth of M. smegmatis wt and knockout strains in minimal 7H9 media supplemented with isovalerate (IVAL). The wt is in green; ΔaccD1-ΔaccA1 is in red; ΔaccD2-ΔaccA2 (control) is in blue. Error bars are in black. (B) Rescue of the M. smegmatis growth phenotype observed in isovalerate-containing minimal media by complementing ΔaccD1-ΔaccA1 (blue line) with the M. tuberculosis homolog of AccD1-AccA1 (red line). The wt is in green. Average values for the culture density were calculated from four replicates.

*M*. *smegmatis* metabolizes leucine, but cannot actively grow on leucine as the sole carbon source (S7 Fig.). Therefore, we studied the growth behavior of wt *M*. *smegmatis* and the Δ*accD1-*Δ*accA1* and Δ*accD2-*Δ*accA2* strains in media containing isovalerate as the sole carbon source. Isovalerate is an intermediate of leucine degradation upstream of MCC, which leads to the accumulation of methylcrotonyl-CoA in Δ*accD1-*Δ*accA1* comparable to leucine feeding. While growth of the Δ*accD2-*Δ*accA2* strain was similar to wt, Δ*accD1-*Δ*accA1* did not grow on isovalerate (Fig. 5A). These data directly illustrate the involvement of the AccD1-AccA1 complex in the catabolism of isovalerate and, hence, in the metabolic pathway of leucine degradation.

To demonstrate the specificity of the observed growth defect of Δ*accD1*-Δ*accA1*, we complemented the Δ*accD1-*Δ*accA1* strain with the homologous *M*. *tuberculosis accD1-accA1* genes. Growth of the Δ*accD1*-Δ*accA1* strain on isovalerate was rescued by *M*. *tuberculosis accD1-accA1* (Fig. 5B). These findings confirm that the MCC activity is specific to the AccD1-AccA1 complex and that its function is conserved in both *M*. *smegmatis* and *M*. *tuberculosis*.

### AccD1-AccA1 forms a dodecameric 6:6 YCC holo complex

**Fig 6:**
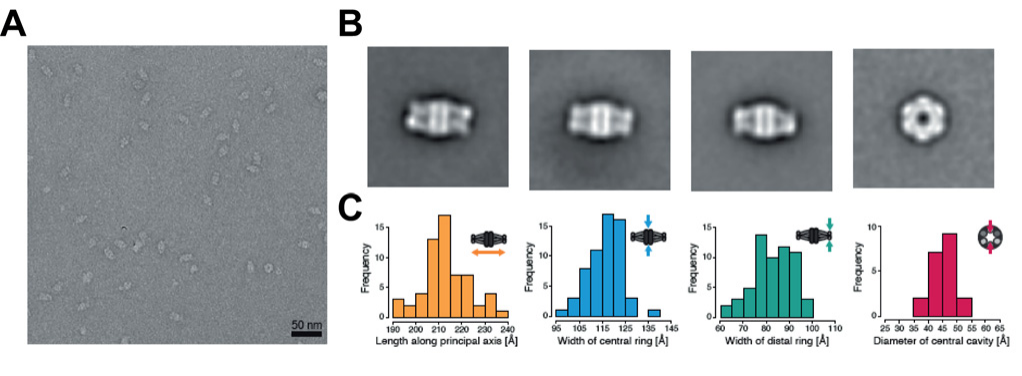
Architecture of the *Mycobacterium tuberculosis* AccD1-AccA1 complex. (A) Electron micrograph of negatively stained AccD1-AccA1 complex. (B) Selected class averages representing the most abundant views of the AccD1-AccA1 complex. (C) Histograms of particle dimensions. The individual dimensions are schematically indicated in relation to the particle appearance in the class averages.

The YCC complex of *Pseudomonas aeruginosa* is currently the only structurally characterized prokaryotic complex with reported MCC substrate specificity [2]. We were hence eager to determine the structural features of the identified mycobacterial MCC and compare it to the reported quaternary assembly of the *P*. *aeruginosa* MCC. To this end, we carried out electron microscopy (EM) studies on the recombinant AccD1-AccA1 complex of *M*. *tuberculosis*. The homogeneity of the complex in electron micrographs confirms the existence of a stable assembly with a defined stoichiometry (Fig. 6A). From a total of 20 class averages (S8 Fig.), we selected four representative classes for further interpretation. A three-layered structure could be identified that is consistent with 32-point group symmetry (Fig. 6B). By using available information from related non-mycobacterial YCCs [2], we assigned the three tiers to be composed of a central MC β-subunit hexamer with internal 32 symmetry, which is flanked on either side by distal BC α-subunit trimers, leading to an overall α_3–_β_6–_α_3_ arrangement of the complete holo complex. The estimated length of the complex along the three-fold axis is 217 ± 10 Å, and the diameter of the inner cavity observed is 42 ± 5 Å. The width of the central β-subunit tier is 117 ± 7 Å, and that of the two peripheral α-subunit layers is 83 ± 9 Å (Fig. 6C, Table 1). Although the density distribution in particle top views suggests that the complex encloses a cavity along the three-fold axis, different top views observed in our class averages (S8 Fig.) suggest conformational variability at the particle ends. As discussed in more detail below, these structural parameters point to a distinct MCC quaternary assembly type that is different from generic YCC holo complexes that catalyze α-carbon carboxylation reactions.

**Table 1:** Dimensions of AccD1-AccA1 and YCC complexes with known high-resolution structures.

	Structure ^a^	Length along principal axis (C3) ^b^	Width of central β-subunit assembly ^b^	Width of outerα-subunit assembly ^b^	Diameter of inner cavity ^b^
*M*. *tuberculosis MCC* ^*c*^	EM	217 ± 10 Å	117 ± 7 Å	83 ± 9 Å	42 ± 5 Å
*P*. *aeruginosa* MCC	3u9s	190 Å	110 Å	75 Å	40 Å
Hybrid PCC ^d^	3n6r	150 Å	120 Å	130 Å	30 Å

^a^ For available X-ray structures, the entry from the Protein Data Bank are given.

^b^ Length measurements for *M*. *tuberculosis* MCC and *P*. *aeruginosa* MCC are from coordinate sets of holo complexes.

^c^ AccD1-AccA1, this contribution. For further details, see Text and Fig. 6.

^d^ *Ruegeria pomeroyi* (BC α-subunit) / *Roseobacter denitrificans* (CT β-subunit).

## Discussion

To unravel functionality in the highly redundant YCC complexes of *M*. *tuberculosis*, we performed a systematic proteomics analysis by which we identified and validated nine binary protein/protein interactions and four acyl-CoA carboxylase holo complexes. Based on these data, we selected the YCC complex AccD1-AccA1 of hitherto unknown function for further mechanistic and functional characterization. We discovered by a combined genetics, metabolomics and structure-oriented approach that this complex is involved in leucine catabolism. It functions as an MCC catalyzing the γ-carboxylation of an unsaturated fatty acid CoA ester rather than the α-carboxylation of saturated fatty acid esters that are catalyzed by various other YCCs. Our and previous data suggest that these two types of reactions are associated with distinct structural requirements. To the best our knowledge, this is the first characterization of a mycobacterial MCC at the molecular level.

The importance of leucine biosynthesis in mycobacteria has been demonstrated previously by generating an *M*. *tuberculosis* leucine auxotroph with an attenuated infection profile *in vivo* [22]. However, very little has been published about the specific role of branched amino-acid catabolism in mycobacteria. Hence, we became interested in whether the genes *accD1* and *accA1* are embedded in a genetic environment that would further support an involvement in this process. In closely related mycobacteria such as *M*. *tuberculosis*, *M*. *avium* and *M*. *smegmatis*, the organization of the *accD1-* and *accA1-*containing operon covering coded genes Rv2498c to Rv2504c (*citE-scoA* operon) is conserved, except for Rv2503c to Rv2505c (S1 Fig.). Interestingly, this pattern of conservation extends into the neighboring *pdhABC* operon that includes genes Rv2495c to Rv2497c, which encode a lipoamide dehydrogenase-dependent keto acid dehydrogenase complex [23]. This complex acts upstream in branched amino-acid catabolism and one of its key functions is thought to be similar to that associated with genes from the *citE-scoA* operon, namely to avoid accumulation of toxic levels of branched-chain amino keto acids and to provide an alternative pool for acetyl-CoA biosynthesis. Mechanistically, lipoylation of the keto acid dehydrogenase complex by lipoamide dehydrogenase (LPD) is analogous to the requirement for the biotinylation of YCC enzymes.

Remarkably, in addition to the conserved operon organization, the expression of most genes (Rv2495c to Rv2501c) of the *citE-scoA* operon and the *pdhABC* operon are strongly correlated (for further details see Operon Correlation Browser (www.broadinstitute.org/annotation/tbdb/operon/), suggesting that the effects observed are coupled. This observation has been confirmed recently by data from *M*. *smegmatis* showing that the two operons form one regulon that is under the control of the TetR-like repressor *bkaR*, which is encoded next to the *citE-scoA* operon by *Rv2506* in *M*. *tuberculosis* and *MSMEG_4718* in *M*. *smegmatis* [24]. Data on the *pdhABC* operon show that the genes it contains crucially contribute to mycobacterial virulence [23] and it will be interesting to unravel whether the argument can also be extended to the genes involved in the *citE-scoA* (Rv2498c-Rv2505c) operon. Taking our data and previously published findings together, it becomes evident that the MCC-encoding genes *accD1* and *accA1* are embedded into a genetic environment that feeds amino-acid degradation into the production of metabolites that ultimately provide an alternative pool for acetyl-CoA. At this point, however, we cannot exclude that the physiological purpose of such an alternative pool of acetyl-CoA in *M*. *smegmatis* and M. *tuberculosis* may serve different purposes. Therefore, the precise physiological role of leucine catabolism in *M*. *tuberculosis* still remains to be elucidated.

Previous structural data on several YCCs have shown that, despite the different chemical requirements for α-carboxylation and γ-carboxylation, the conformation of the CT active site is conserved and deeply buried in a symmetric CT-CT interface [25,26,27,28]. However, recent structures of two holo propionyl-CoA carboxylase (PCC) and MCC complexes revealed that the overall conformations of the dodecameric complexes are substantially different despite significant global sequence similarities [2,17,29]. The observed differences are because of a swap in the arrangement of the N-terminal and C-terminal domains within the individual CT β-subunits, leading to relocation of the BCCP domain. The overall dimension estimates of the two arrangements are also substantially different and can be distinguished even at low resolution (Table 1). Mechanistically, the reasons for the divergent arrangements, however, are not understood [2].

The dodecameric arrangement of α- and β-subunits in YCCs has been observed in the AccD1-AccA1 and the related MCC of *P*. *aeruginosa*, as well as the chimeric PCC [17,29]. The AccD5-AccA3 complex is presumed to be a dodecamer based on its size estimates from gel filtration data and the β-subunit structure on its own is a hexamer similar to those -subunits in YCC dodecamers [6,25,28]. It would therefore seem that a dodecameric arrangement is common to most bacterial YCCs. However, the *M*. *tuberculosis* AccD6 has a dimeric quaternary structure [30]. The hexameric β-subunits are trimers of dimers, AccD6 being structurally equivalent to one such dimer. A dodecameric arrangement of subunits can therefore not be assumed for all YCC complexes. Interestingly, a new structure of an unusual bacterial single-chain YCC assembles into a homo-hexameric assembly with yet another unrelated overall arrangement [31], indicating that possible combinations for different arrangements may be even more diverse than previously anticipated.

Comparison of the CT β-subunit sequence of the *M*. *tuberculosis* AccD1-AccA1 complex with the sequences used for structure determination of the two known YCC holo complexes reveals 66% sequence identity with the *P*. *aeruginosa* CT subunit of the MCC complex and 33% sequence identity with the *Roseobacter denitrificans* CT subunit of the PCC complex. In structural terms, the set of *M*. *tuberculosis* AccD1-AccA1 class averages in comparison to simulated projections of the MCC (PDB ID 3u9s) and PCC (PDB ID 3n6r) complexes illustrates that the conformation of AccD1-AccA1 closely resembles that of the *P*. *aeruginosa* MCC complex (Fig. 6, S8 Fig.). All molecular dimensions inferred from individual AccD1-AccA1 particles in our electron micrographs are compatible with structural parameters derived from the MCC complex (PDB ID 3u9s) (Table 1). By contrast, the dimension estimates from the structure of the only available PCC complex (PDB ID 3n6r) differ significantly. Thus, our functional findings—identifying AccD1-AccA1 from mycobacteria as an MCC—are supported at the level of structure and sequence.

**Fig 7:**
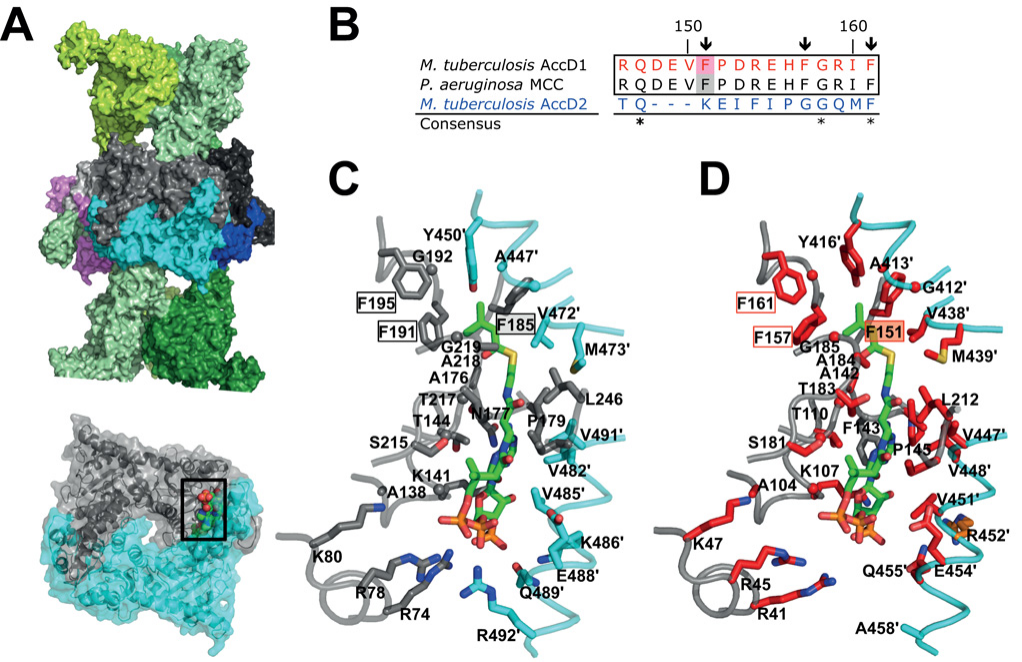
Structural conservation of the *M. tuberculosis* AccD1 CT active site. (A) Overall MCC quaternary arrangement, as determined previously for MCC from P. aeruginosa [17]. The BT subunits of the two distal trimeric tiers are shown in different green shadings. The central hexameric CT subunit assembly is shown in different colors. One dimeric CT subunit assembly in grey/cyan has been depicted in the lower panel and the CT active site formed within the dimeric interface is indicated by sphere presentation of the modeled substrate 3-methylcrotonyl-CoA with atom-specific colors (carbon, green) [17]. (B) Sequence alignment of the predicted M. tuberculosis AccD1 3-methylcrotonyl-CoA substrate-binding segment with the respective sequence segments of the P. aeruginosa MCC and M. tuberculosis AccD2 of unknown function. The residues numbers indicated refer to the AccD1 sequence. Conserved residue positions are indicated with asterisks. Residue positions that are conserved in the two upper confirmed MCC sequences (Phe151, Phe158, Phe161 in M. tuberculosis AccD1) but not in the AccD2 sequence are indicated by arrows. The complete sequence alignment is shown in S9 Fig. (C) Residues that interact with 3-methylcrotonyl-CoA in the P. aeruginosa MCC model [17], as determined with PISA [47], are shown in stick presentation and are labeled. Color codes are as in panel A. (D) Homology model of the M. tuberculosis AccD1 CT active site. Residues that are invariant in an alignment of the CT sequences from P. aeruginosa and M. tuberculosis (S9 Fig.) are in red, demonstrating a very high level of conservation of the AccD1 active site; conserved residues are in orange, non-conserved residues are in grey.

At present, there is no atomic structure of an MCC CT domain in the presence of 3-methylcrotonyl-CoA. However, as the structure of the known MCC holo complex contains CoA in the active site of the CT domain, it was possible to approximately localize the methylcrotonyl-CoA-binding pocket, and this points to the crucial importance of Phe191 in the *P*. *aeruginosa* MCC sequence as one of the key substrate specificity determinants (Fig. 7A) [17]. A sequence alignment with the *M*. *tuberculosis* AccD1 sequence reveals complete conservation of a 16-residue stretch “RQDEVFPDREHFGRIF” flanking the equivalent Phe157 and Phe191 in the *M*. *tuberculosis* and *P*. *aeruginosa* sequences, respectively (Fig. 7B, S9 Fig.). By contrast, this sequence segment is highly divergent in all other YCC CT subunit sequences, implying functional divergence of the AccD1-AccA1 complex. Interestingly, this also applies to the CT AccD2 subunit, which of all the other *M*. *tuberculosis* CT sequences is most closely related in overall sequence to AccD1 (Fig. 7B). Based on these and our functional metabolomics data, we predict that the mycobacterial AccD2-AccA2 complex may have the same overall architecture as the AccD1-AccA1 complex, but differs in YCC substrate specificity (Fig. 7C–D), leaving it an open question how this YCC is involved in mycobacterial metabolism.

Finally, we tried to devise possible structural and functional rationales to explain why the *M*. *tuberculosis* AccD1-AccA1 holo complex fits the conformation of the *P*. *aeruginosa* MCC rather than that of the only available PCC structure known to date. Attempts in modeling the AccD1 and AccA1 sequences onto the two available MCC and PCC holo complex structures, however, did not reveal any substantial energetic differences between the resulting models that could explain why one conformation would be preferred over the other. Therefore, experimental high-resolution structural data of holo YCC complexes will be essential to clarify the role of this helix and other possible factors in defining the overall arrangement of these enzymes.

## Materials and Methods

### Co-immunoprecipitation and mass spectrometry

All YCC genes from *M*. *smegmatis* (S1 Table) were amplified and cloned into a pKW08 mycobacterial tetracycline-inducible vector [32,33]. The amplicons were cloned upstream of the Tobacco Etch Virus (TEF) cleavage site, followed by an Enhanced Green Fluorescent Protein (eGFP) tag, and downstream of the TetR08 promoter. Cloning, expression, and a single-step protein purification protocol followed by LC-MS/MS analysis was applied as described [34]. Proteins were identified by searching the data against the *M*. *smegmatis* protein database (http://cmr.jcvi.org/tigr-scripts/CMR/CmrHomePage.cgi). The database was modified to add randomized sequences of all entries as a control of false-positive identifications during analysis. Fragmentation spectra were searched using the Andromeda search engine integrated into the MaxQuant (v1.3.0.5) platform. Computational data analysis was performed using the Perseus tool (v1.3.0.4, Cox J., Max Planck, 2012). Contaminants and random protein identification were excluded. Proteins that were identified by less than two peptides were excluded from the result list. The proteomics data have been submitted to the Proteomicsdb database (https://www.proteomicsdb.org/proteomicsdb/#projects/4170).

### Creation of knockout strains

*M*. *smegmatis* YCC genes *accD1*, *accA1*, *accD2* and *accA2* were deleted by recombineering [35], creating the double-knockout strains Δ*accD1-* Δ*accA1* and Δ*accD2-* Δ*accA2*. As *accD1*-*accA1* and *accD2-accA2* are each encoded in an operon, both sets of genes could be deleted using a single recombination event. To produce the double knockouts a linear target substrate had to be created. Two 500 bp or 1000 bp fragments, homologous to the up- and downstream regions of the relevant genes, were amplified by PCR using specific primer pairs and subsequently digested with specific restriction endonucleases (S4 Table). The amplicons were ligated into pYUB854 digested with *Kpn*I-*Xba*I or *Afl*II-*Avr*I (for the fragment of the upstream region, depending on the type of restriction sites present) and *Hind*III-*Spe*I (for the fragment of the downstream region) [36]. In this manner, constructs pEN30 and pEN43 were created (S5 Table). To obtain the linear target substrate, pEN30 was digested with *Kpn*I and *Spe*I, and pEN43 with *Afl*II and *Spe*I. The linear DNAs were subsequently transformed into *M*. *smegmatis* mc^2^155 electrocompetent cells expressing the recombinase genes on pJV53 [35] generating hygromycin-resistant recombinants. The hygromycin-resistance cassette was removed using δγ-resolvase expressed on pGH542 generating the unmarked deletion strains [37].

Genomic DNA of potential mutants was isolated [35] and Southern hybridization was used to confirm the absence of the particular gene (S10 Fig.). Genomic DNA (5 μg) for Southern blot analysis was digested with the appropriate enzymes, separated on a 0.9% agarose gel and transferred to a positively charged nylon membrane (Roche). DNA-probe labeling, hybridization and detection were performed using the digoxigenin high prime DNA labeling and detection starter kit 1 as described by the manufacturer (Roche).

### Lipid analysis of *M*. *smegmatis* strains

Cells from *M*. *smegmatis* mc^2^ 155 and the *M*. *smegmatis* knockout strains Δ*accD1-*Δ*accA1* and Δ*accD2-*Δ*accA2* were grown for 24h on 7H9 medium supplemented with 0.2% glycerol and 5% OADC at 37°C. Wet cell pellets were treated with mixtures of CHCl_3_/CH_3_OH (1:2, 1:1, 2:1) to yield total lipid extracts (TLE) that were pooled and dried under vacuum. Fatty acids and mycolic acids were isolated by treating the bacterial cells or TLE with a 5% KOH solution in methanol/toluene (8:2 v/v) under reflux for 8 hours. After acidification, fatty acids were extracted with diethyl ether and methylated with an ethereal solution of diazomethane. The fatty acid methyl ester (FAME) and mycolic acid methyl ester patterns of the strains were determined by analytical thin-layer chromatography (TLC) on Silica Gel 60 (Macherey-Nagel). For fatty acid and mycolic acid labeling during bacterial growth, cultures of wt and the knockout strains were grown for 24 hours and then centrifuged for 15 minutes at 3000 x g at 4°C. The bacterial pellets were suspended in fresh 7H9/glycerol medium and incubated for 30 min at 37°C before addition of 1 μCi/μl of [1–^14^C] acetic acid (56.6 mCi/mmole, Perkin Elmer). After 1 hour incubation with shaking (200 rpm) the bacteria were harvested by centrifugation and the cell pellet was saponified and treated as above. Radio-labeled methyl esters were resolved in CH_2_Cl_2_ as running solvent at room temperature and visualized by exposing the TLC to Fujifilm imaging plate prior to phosphorImager detection (Typhoon Trio GE Healthcare).

### Metabolic sampling and untargeted CoA-profiling

Bacterial strains were pre-cultured overnight in modified 7H9 medium containing 0.2% glycerol and 0.025% tyloxapol at 37°C, 300 rpm. We defined modified 7H9 media as media containing all constituents of 7H9 with the exception of glutamic acid, as this could serve as a C-source and could affect growth data collected in selective media to which we have added a specific C-source. Preculture was inoculated into 25 mls of the same culture media to an OD_600_ of 0.015 and incubated at 37°C under constant shaking at 300 rpm. Metabolic samples were collected during mid-exponential growth phase (OD_600_ = 0.6 to 1.0). Sampling of metabolites followed the same principle as previously described [19]. In brief, a culture volume corresponding to a biomass of 3 ml bacterial culture at an OD_600_ of 1.0 was rapidly harvested through 0.45 μm filters by vacuum filtration and washed with 75 mM ammonium carbonate buffer (pH 6.6). The entire harvesting and washing procedure took less than 20 s. Filters were immediately submerged in 60% ethanol at 78°C for 2 min and then shock-frozen in liquid nitrogen. Samples were dried at 30°C in a SpeedVac equipped with a cooling trap at -85°C. The dried extracts were stored at -80°C until further analysis by LC-MS/MS. The samples were re-suspended in 100 μl water and 10 μl were injected for LC-MS/MS analysis. Untargeted profiling of the CoA thioesters was performed as described previously [19].

### Culture media shift experiments

Bacterial strains were cultured and sampled for metabolic measurements during mid-exponential growth phase as described above. Bacteria of the remaining cultures were centrifuged at 3000 x g for 2 min, cells were washed with 15 ml of modified 7H9 medium supplemented with 0.025% tyloxapol and after centrifugation re-suspended in 15 ml of modified 7H9 supplemented with 0.025% tyloxapol and containing 0.4% leucine. Metabolic samples were collected after 1 hour of additional incubation at 37°C under constant shaking at 300 rpm.

### Quantification of CoA thioesters by SRM assays

The same chromatographic separation protocol that was used for the untargeted profiling of CoA thioesters was applied for the quantification of CoA thioesters by selected reaction monitoring (SRM) using LC-MS/MS. Ion pairing-reverse phase chromatography at ultrahigh pressure using a Waters Acquity UPLC (Waters Corporation, Milford, MA, United States) with a Waters Acquity HSS T3 column with dimensions 150 mm × 2.1 mm ×1.8 μm (Waters Corporation, Milford, MA, United States) at 40°C was used. A gradient of mobile phases A (10 mM tributylamine, 15 mM acetic acid at pH 5.0, 5% (v/v) methanol) and B (2- propanol) was applied. This yielded in the following settings: initial conditions: 0% B, 0.4 mL/min; 0.5 min: 0% B, 0.4 mL/min; 1.5 min: 12% B, 0.4 mL/min; 10 min: 27.5% B, 0.25 mL/min; 20 min: 90% B, 0.15 mL/min; 25 min: 90% B, 0.15 mL/min; 28 min: 0% B; 0.15 mL/min; 35 min: 0% B; 0.4 mL/min. The fragment specific for coenzyme A with a m/z = 408 at negative operation mode of the mass spectrometer was used for the SRM assays on a Thermo TSQ Quantum Ultra triple quadrupole instrument (Thermo Fisher Scientific, Waltham, MA, United States). The other SRM parameters were set to 140 V and 39 (arbitrary unit) for the tube lens and the collision energy, respectively. Dried metabolic samples were re-suspended in 100 μl water and 10 μl were injected for LC-MS/MS analysis. Concentrations of CoA thioesters were quantified using dilutions of analytical standards between 14 and 0.45 μM, if commercially available. Estimated purity of the compounds according to the manufacturer was taken into account for the calculations.

### *M*. *smegmatis* growth assays

Wt and knockout strains of *M*. *smegmatis* were pre-cultured from frozen glycerol stocks in 7H9 ADC culture medium to mid-exponential growth phase (37°C, 300 rpm). The bacteria were centrifuged at 3000 x g for 2 min, washed with one culture volume (3 ml) of modified 7H9 medium, centrifuged again and re-suspended in culture medium containing the carbon source to be tested (3 ml). The bacteria were incubated at 37°C under constant shaking (300 rpm) for another 24 h. These cultures were used to inoculate a Nunc 48-well plate (Nunclon delta SI, 500 μl per well) with the according culture medium. A plate reader (Tecan Infinite M200) was used to incubate the plate at 37°C under constant orbital shaking and to monitor the culture density by measuring the OD_600_ every 10 min (25 flashes, 9 nm bandwidth). Average values for the culture density were calculated from four replicates.

The same procedure was applied for the complementation of the knockout strain. However, in addition 0.001% acetamide was added to induce protein expression for the second pre-culture and the culturing in 48-well plates. The creation of the complementation plasmid is described in a subsequent section.

### Creation of expression and complementation construct

The sequence of *M*. *tuberculosis* AccD1 (Rv2502c) and AccA1 (Rv2501c) are encoded in an operon, with only a 4 base-pair intergenic region whereas *M*. *tuberculosis* AccD2 (RV0974c) and AccA2 (Rv0973c) are encoded in one operon lacking an intergenic region S1 Fig.). Co-expression of proteins encoded in operons has been a successful strategy for the production of mycobacterial protein complexes [38] and was used to express these YCC complexes. The entire coding sequence of *accD1* and *accA1*, including the intergenic region, was amplified using primers MTE01 and MTE02 from *M*. *tuberculosis* H37Rv genomic DNA (S4 Table). The primers MTE03 and MTE04 were used to amplify the entire accD2-accA2 region. Both the amplicon of accD1-accA1 and of accD2-accA2 were directionally cloned into the mycobacterial expression vector pMyNT, between the restriction enzyme sites *Nco*I and *Hind*III, immediately downstream of a histidine tag (S5 Table). Only the AccD protein was His-tagged. The constructs were sequenced to verify insertion of the amplicon. The construct pMyNT D1A1, encoding *accD1-accA1*, was used in the *M*. *smegmatis* complementation experiment and for production of recombinant AccD1-AccA1 for biochemical characterization. The construct pMyNT D2A2, encoding accD2-accA2, was used to produce recombinant AccD2-AccA2.

### Protein production and purification

Constructs were transformed into *M*. *smegmatis* mc^2^155 *groEL1ΔC* to improve protein yield during purification [39]. Transformation was performed as described [38]. AccD1-AccA1 and AccD2-AccA2 were produced as follows. Transformants were cultured in 7H9 medium supplemented with 0.2% glucose, 0.2% glycerol, 0.05% Tween 80, and 0.1 nM hygromycin. A starter culture of 50 ml volume was grown for 2 days at 37°C. One percent of this starter served as an inoculum for a larger 500 ml culture, which was incubated under the same conditions. Protein expression was induced with acetamide, at a final concentration of 30 mM, when cultures had reached an optical density of 3.5 measured at 600 nm. The cultures were incubated for a further 20 hours. Thereafter the cells were collected by centrifugation at 3000 x g and 4°C for 60 min. Culture pellets were resuspended in 25 ml buffer containing 50 mM Tris-HCl pH 7.2, 300 mM NaCl and complete protease inhibitor cocktail (Serva) and stored at -20°C until needed. The production of the AccD5-AccA3 complex has been reported elsewhere [39].

Cells were lysed by sonication, following the addition of 1 mg/ml DNase I (Serva) was added. Cells were sonicated using a Bandelin Sonoplus HD3200 sonicator set to pulse with an on-off cycle of 0.3 sec—0.7 sec and an amplitude of 45% for a total of 3 min. Sonication was repeated 3 times with a 3 min period between sonication events to prevent the sample from overheating. The sample was cooled on ice throughout. The lysate was centrifuged at 38,700 x g for 1 hour. The resultant cleared lysate was then filtered through a 0.22 μm filter and the filtrate loaded onto a 5 ml nickel-nitrilotriacetic acid affinity column (Qiagen). The affinity column was washed with 4 column volumes of 50 mM Tris-HCl pH 7.2, 100 mM NaCl, 20 mM imidazole and protein eluted with 50 mM Tris-HCl pH 7.2, 100 mM NaCl, 250 mM imidazole. Affinity eluates containing the protein were pooled, concentrated and loaded onto a Superose 6 10/300 GL size-exclusion column (GE Healthcare), which had been pre-equilibrated with 50 mM Tris-HCl pH 7.2, 100 mM NaCl, 1 mM dithiothreitol. Protein fractions from the size-exclusion column were collected and analyzed by electrophoresis using gels that had a 10% polyacrylamide separating gel. The identity of the proteins was confirmed by peptide-mass fingerprinting performed by the EMBL Proteomics Core Facility, Heidelberg, Germany. A sample of pure protein complex was re-loaded onto the analytical Superose column to estimate its molecular size. A high molecular weight standards kit was used to calibrate the column (Amersham).

### Enzyme assay

The assay we used to measure CT activity is a spectrophotometric-coupled enzyme assay [40]. The ATP-dependent carboxylation of acyl-CoA substrates by the YCC is coupled to the ADP-dependent dephosphorylation of phosphoenolpyruvate and the subsequent NADH reduction of the lactate product, catalyzed by pyruvate kinase and lactate dehydrogenase, respectively. The latter reaction results in the consumption of NADH, which is monitored at 340 nm. Spectrophotometric data were recorded using a PowerWaveX Select spectrophotometer (Bio-Tek Instruments) and Greiner Bio-one UV-transparent, flat-bottom 96-well plates. Data were recorded using KC4 Kineticalc version 3.01 (Bio-Tek Instruments) and analyzed using GraphPad Prism 5 version 5.03 (Graphpad Software Inc.). The reaction mixture contained 7 U pyruvate kinase, 10 U lactate dehydrogenase, 0.5 mM phosphoenolpyruvate, 0.2 mM NADH, 5 mM MgCl_2_, 100 mM K_2_HPO_4_ pH 7.6, 3 mM ATP, 50 mM NaHCO_3_, 0.3 mg/ml bovine serum albumin and varying concentrations of 3-methylcrotonyl-CoA substrate between 1.5 mM and 25 mM. Both the reaction mixture and protein samples were incubated at 30°C for 2 min prior to the start of the assay. The reaction was started upon addition of purified protein to the reaction mixture and consumption of NADH then measured every 30 sec for 1 hour at 30°C. The enzyme concentration-dependent carboxylation of the different substrates had been previously assessed and the concentration of enzyme at which first-order kinetics are maintained was 3 μM. This concentration of AccD1-AccA1 was used in all experiments.

The experiment comparing AccD1-AccA1 and AccD5-AccA3 activity used the same reaction as described above, with the exception that the protein concentration was fixed at 0.6 μM and the concentration of 3-methylcrotonyl-CoA was 25 mM. Carboxylation of the substrate was followed for 15 min.

### Electron microscopy

Purified AccD1-AccA1 complex at a concentration of approximately 0.1 mg/ml in 50 mM HEPES (pH 7.2), 100 mM NaCl, 1 mM DTT, 2 mM MgCl_2_ was applied to the carbon-side of a carbon-mica interface before it was transferred to an EM grid and stained with 2% uranyl acetate. Images were recorded under low-dose conditions at ambient temperature using a Philips CM200 FEG microscope operated at 200 kV. Images were acquired at a nominal 27,500× magnification resulting in a pixel size of 6.2 Å at the specimen level. All micrographs were recorded on a bottom-mounted GATAN 794 1 x 1K CCD Camera (GATAN Inc., Pleasanton, CA).

Semi-automatic particle selection was performed using EMAN2’s e2boxer [41]. A total of 5,837 particles were selected from 100 1K x 1K CCD images and windowed into 80 × 80 pixel particles that were subsequently low-pass filtered at 25 Å resolution and normalized. Initial translational alignment was performed with respect to the rotationally averaged total sum of the particle images. An initial reference-free classification step with the multivariate statistical analysis (MSA) procedure [42] was followed by five iterative rounds of multi-reference alignment to improve translational and rotational alignment of the particle images and subsequent MSA classification, which resulted in 20 stable classes.

Simulated projections of *Pseudomonas aeruginosa* MCC (PDB ID 3u9s) were generated using the SPIDER processing package [43]. To obtain a uniform distribution of orientations, projections of a simulated map at 25 Å resolution were generated in 5° angular increments. For visual comparison we aligned the individual projections to the AccD1-AccA1 class averages.

### Homology modeling

Homology modeling of the AccD1-AccA1 complex was performed with MODELLER-9v7 [44]. Based on multiple sequence alignment of *M*. *tuberculosis* AccA1 and AccD1 to the α- and β-subunits of PaMCC (PDB ID 3u9s) and PCC (3n6r) using MUSCLE (https://www.ebi.ac.uk/Tools/msa/muscle/), four sets of five homology models each were built using monomeric α- and β-subunits of PaMCC (PDB ID 3u9s) or PCC (PDB ID 3n6r) as template structures for AccA1 and AccD1, respectively. The resulting models were ranked according to the DOPE statistical potential score [45]. The best ranking models were used for generation of the holo complexes by applying symmetry transformations and subsequently energy minimized in PHENIX [46].

## Supporting Information

S1 Table Sequence identities of aligned AccD CT β-subunit sequences. Right-most column: sequence similarity expressed in % identity between *M*. *tuberculosis* YCC β-subunits and *P*. *aeruginosa* MCC β-subunit; bottom row: sequence similarity expressed in % identity between *M*. *smegmatis* YCC β-subunits and *P*. *aeruginosa* MCC β-subunit; diagonal (in bold): sequence similarity expressed in % identity between homologous *M*. *tuberculosis* and *M*. *smegmatis* YCC β-subunits.
(DOC)

S2 Table Complete list of mass spectrometry data on YCC pull-down experiments. The table columns represent the number of peptides identified, coverage and intensities of peptides from proteins identified in pull-down experiments with different baits that are labeled in headers.
(XLSX)

S3 Table MALDI-TOF mass spectrometry data of the individual types of mycolic acid methyl esters [48]. (DOC)

S4 Table List of primers. Restriction sites used for cloning are presented in bold face. CTTAAG = AflII, CCTAGG = AvrII, AAGCTT = HindIII, ACTAGT = SpeI, TCTAGA = XbaI, GGTACC = KpnI, CCATGG = NcoI. CCATGG = PstI, Up and Down signify the fragments homologous to the upstream and downstream region of the particular gene(s) that needed to be deleted.
(DOC)

S5 Table Plasmids and constructs. (DOC)

S1 Fig Genes *accD1* and *accA1* belong to the *citE-scoA* operon in *M. tuberculosis*. Structure of the *M*. *tuberculosis citE-scoA* operon (Rv2498c to Rv3504c) and the preceding *pdhC-pdhA* operon (Rv2495c to Rv25497c) and related operon structures in *M*. *avium*, *M*. *smegmatis*, *R*. *sp*. *RHA1*, and *S*. *avermitilis*. Homologous genes are shown in identical colors and are annotated with their standard gene identifiers. In addition, for all those *M*. *tuberculosis* genes with predicted or known function, additional functional gene codes are also presented. The genes Rv2591c (*accD1*) and Rv2502c (*accA1*) from *M*. *tuberculosis*, the subject of this contribution, and homologous genes from other shown organisms are boxed.
(TIF)

S2 Fig Evidence for AccD2-AccA2 holo complex formation by size-exclusion chromatography and SDS-PAGE (inset). (TIF)

S3 Fig Growth of the *M. smegmatis* Δ*accD1*-Δ*accA1* and Δ*accD2*-Δ*accA2* strain in 7H9 medium. Batch cultures of each strain were grown in triplicate at 37°C and the cell density monitored by measuring the optical density of the culture at 600nm every 4 hours. The wt is in green, Δ*accD1-*Δ*accA1* is in red, Δ*accD2-*Δ*accA2* is in black.
(TIF)

S4 Fig Lipid composition of wt *M. smegmatis* and the knockout strains. TLC analysis of labeled lipids (FAMEs and MAMEs) of wt and the knockouts strains after incubation with [1–^14^C]acetic acid. FAMEs: fatty acid methyl esters, MAMEs: mycolic acid methyl esters (with the three types of *M*. *smegmatis* mycolic acids alpha, alpha’ and epoxy). The expected migration of meromycolic acid- and alkylmalonic acid-methyl esters (ME) is indicated on the TLC. TLC was developed with dichloromethane and visualization performed by phosphorImager.
(TIF)

S5 Fig Detection of the AccD2-AccA2 complex reaction product. LC-MS/MS analysis of CoA thioester in samples containing putative substrate, 3-methylcrotonyl-CoA, and the enzyme AccD1-AccA1 as determined under the assay conditions described under Materials and Methods. After 30 min of incubation the assay mixture was snap-frozen and the CoA thioesters were analyzed by LC-MS/MS as described. Besides the substrate peak the production of an additional CoA thioester was detected in the sample containing the native enzyme (upper panel) but it was absent in the sample containing heat-denatured (10 min at 95°C) enzyme (lower panel). This CoA thioester corresponds to methylglutaconyl-CoA with an m/z of 892 Da of its deprotonated form. The mass difference of 44 Da between 3-methylcrotonyl-CoA and methylglutaconyl-CoA reflects the carboxylation reaction.
(TIF)

S6 Fig AccD1-AccA1 activity on intermediates of branched chain amino acid metabolism *in vivo*. Nutritional shifts from cultures grown on glycine as sole carbon source to a culture medium containing leucine, 4-methyl-2-oxopentanoate, isoleucine, 3-methyl-2-oxopentanoate, valine, or 3-methyl-2-oxobutanoate as sole carbon source. One hour after the nutrient shift, intracellular metabolites were extracted and the CoA thioesters were analyzed by LC-MS/MS as described in the manuscript. The shift to either leucine or 4-methyl-2-oxopentanoate led to a striking accumulation of 3-methylcrotonyl-CoA in the Δ*accD1-*Δ*accA1* strain. However, the shift to either valine or 3-methyl-2-oxobutanoate did not lead to the accumulation of methylacrylyl-CoA in the same strain, as no peak with an m/z of 834 Da in negative mode was significantly different in the Δ*accD1-*Δ*accA1* strain compared to the *wt* strain. The shift to either isoleucine or 3-methyl-2-oxopentanoate did not lead to an accumulation of 2-methylcrotonyl-CoA with an m/z of 848 Da in the Δ*accD1-*Δ*accA1* strain, as one would expect if AccD1-AccA1 were involved in the degradation of isoleucine. In conclusion, these results demonstrate that the principle role of AccD1-AccA1 *in vivo* is the carboxylation of 3-methylcrotonyl-CoA to methylglutaconyl-CoA as part of the leucine degradation pathway.
(TIF)

S7 Fig Growth of wt *M. smegmatis* in medium supplemented with either glucose (green line) or leucine (red line) as sole carbon source. Average values for the culture density were calculated from four replicates.
(TIF)

S8 Fig *M. tuberculosis* AccD1-AccA1 class averages in comparison to simulated projections of *P. aeruginosa* MCC (PDB ID 3u9s) and the hybrid *Ruegeria pomeroyi/Roseobacter* PCC (PDB ID 3n6r). Simulated projections were generated from random orientations with five degree Euler intervals. Projections with highest correlation to selected AccA1-AccD1 class averages are shown in their corresponding orientation. The data demonstrate that the overall conformation of AccD1-AccA1 resembles the MCC complex and is substantially different from that of PCC.
(TIF)

S9 Fig Multiple alignment of CT sequences. AccD1 from *M*. *tuberculosis* (O06165_MYCTU), MCC from *P*. *aeruginosa* (Q9I297_PSEAE), and AccD2 from *M*. *tuberculosis* (0T826_MYCTU). Residue numbers refer to the *M*. *tuberculosis* AccD1 sequence. The *M*. *tuberculosis* AccD2 sequence, which is likely to have different substrate specificity (for details, see text), is shown in blue. In the consensus line, invariant and conserved residues positions are marked by “*” and “:”, respectively. Residues that are involved in binding to 3-methylcrotonyl-CoA within the CT dimer interface of the substrate model of the MCC holo complex *P*. *aeruginosa* [17] are highlighted in grey and cyan, matching the color scheme of Fig. 7. The sequence motif “RQDEVFPDREHFGRIF” (residues 146–161 of the *M*. *tuberculosis* AccD1 sequence), which is invariant in *M*. *tuberculosis* AccD1 and *P*. *aeruginosa* MCC but divergent in *M*. *tuberculosis* AccD2, is boxed (*cf*. Fig. 7). Phe151, Phe157 and Phe161 (*M*. *tuberculosis* AccD1 numbering scheme) are critical in determining 3-methylcrotonyl-CoA binding specificity and are indicated by arrows.
(TIF)

S10 Fig Southern blot analysis of knockout strains verifying the deletion of the relevant genes. (*A*) Analysis of Δ*accD1-*Δ*accA1* and (*B*) Δ*accD2-*Δ*accA2*, performed with genomic DNA of *M*. *smegmatis* mc^2^155 carrying pJV53 (lane 1), correct hygromycin-resistant mutants (lane 2) and correct unmarked deletion mutants (lane 3; a/b when more than one). The *Hin*dIII-*Spe*I digested fragment of pEN30 was used as a probe for Δ*accD1-*Δ*accA1* and the *Afl*II-*Avr*II of pEN43 for Δ*accD2-*Δ*accA2*. The genomic DNA was digested with restriction endonucleases shown in the figure. (M) DNA marker VII, digoxigenin labeled (Roche).
(TIF)
