# Supplementary material for: Characterization of the Mycobacterial Acyl-CoA Carboxylase Holo Complexes Reveals Their Functional Expansion into Amino Acid Catabolism
Source: PLoS Pathog. 2015 Feb 19;11(2):e1004623. doi: 10.1371/journal.ppat.1004623 (PMC4347857; doi:10.1371/journal.ppat.1004623)
Supplement: S1 Table — Right-most column: sequence similarity expressed in % identity between M. tuberculosis YCC β-subunits and P. aeruginosa MCC β-subunit; bottom row: sequence similarity expressed in % identity between M. smegmatis YCC β-subunits and P. aeruginosa MCC β-subunit; diagonal (in bold): sequence similarity expressed in % identity between homologous M. tuberculosis and M. smegmatis YCC β-subunits. (DOC) [file ppat.1004623.s001.doc]

|  |  | ***M. smegmatis*** | | | | | |  |
| --- | --- | --- | --- | --- | --- | --- | --- | --- |
|  |  | AccD6 | AccD5 | AccD4 | AccD3 | AccD2 | AccD1 | MCC(PA) |
| ***M. tuberculosis*** | AccD6^a^ | **89** | 42.0 | 35.5 | 19.1 | 31.2 | 29.9 | 30.1 |
|  | AccD5^b^ | 42.9 | **87** | 48.3 | 20.7 | 31.3 | 32.9 | 32.5 |
|  | AccD4^c^ | 34.2 | 49.2 | **81** | 19.8 | 28.4 | 31.3 | 29.5 |
|  | AccD3^d^ | 19.5 | 21.8 | 21.5 | **79** | 18.7 | 18.8 | 19.0 |
|  | AccD2^e^ | 28.5 | 30.5 | 29.2 | 20.7 | **88** | 48.8 | 53.7 |
|  | AccD1^f^ | 30.6 | 33.3 | 32.3 | 17.1 | 51.5 | **84** | 66.5 |
|  | MCC(PA)^g^ | 29.8 | 29.4 | 32.6 | 18.2 | 54.8 | 68.0 | – |

^a^ AccD6: *M. tuberculosis*, Rv2247, PCC6_MYCTU; *M. smegmatis*, Msmeg4329, A0R0B6_MYCS2.

^b^ AccD5: *M. tuberculosis*, Rv3280, PCC5_MYCTU; *M. smegmatis*, Msmeg1813, A0QTE7_MYCS2.

^c^ AccD4: *M. tuberculosis*, Rv3799c, L7N5B4_MYCTU; *M. smegmatis*, Msmeg6391, A0R616_MYCS2.

^d^ AccD3: *M. tuberculosis*, Rv0904, H6S9R6_MYCTU; *M. smegmatis*, Msmeg5642, A0R3Y8_MYCS2.

^e^ AccD2: *M. tuberculosis*, Rv0974c, L0T826_MYCTU; *M. smegmatis*, Msmeg5492, A0R3J2_MYCS2.

^f^ AccD1: *M. tuberculosis*, Rv2501c, O06165_MYCTU; *M. smegmatis*, Msmeg4717, A0R1D9_MYCS2.

^g^ MCC(PA): P. *aeruginosa,* Q9I297_PSEAE.
