# Supplementary material for: Characterization of the Mycobacterial Acyl-CoA Carboxylase Holo Complexes Reveals Their Functional Expansion into Amino Acid Catabolism
Source: PLoS Pathog. 2015 Feb 19;11(2):e1004623. doi: 10.1371/journal.ppat.1004623 (PMC4347857; doi:10.1371/journal.ppat.1004623)
Supplement: S3 Table — (DOC) [file ppat.1004623.s003.doc]

| **MAME** | **C atoms^a^** | 74 | 75 | 76 | 77 | 78 | 79 | 80 | 81 |
| --- | --- | --- | --- | --- | --- | --- | --- | --- | --- |
| **α** | WT | 1118 | 1132 | 1146 | **1160** | 1174 | **1188** | 1202 |  |
|  | Δ*accD1-*Δ*accA1* | 1118 | 1132 | 1146 | **1160** | 1174 | **1188** | 1202 |  |
|  | Δ*accD2-*Δ*accA2* | 1118 | 1132 | 1146 | **1160** | 1174 | **1188** | 1202 |  |

|  | **C atoms** | 62 | 64 |  |
| --- | --- | --- | --- | --- |
| **α’** | WT | **951** | **979** |  |
|  | Δ*accD1-*Δ*accA1* | **951** | **979** |  |
|  | Δ*accD2-*Δ*accA2* | **951** | **979** |  |

|  | **C atoms** | 74 | 75 | 76 | 77 | 78 | 79 | 80 | 81 |
| --- | --- | --- | --- | --- | --- | --- | --- | --- | --- |
| **Epoxy** | WT |  |  |  |  | **1190** | 1204 | **1218** |  |
|  | Δ*accD1-*Δ*accA1* |  | 1148 | 1162 | **1176** | **1190** | **1204** | **1218** |  |
|  | Δ*accD2-*Δ*accA2* |  | 1148 | 1162 | **1176** | **1190** | **1204** | **1218** | 1232 |

^a^ Values represent the molecular masses of MAME with sodium adduct [M+Na]^+^ and refer to the total carbon atom numbers of the free acid. The mass values of the major homologues representing at least 30-40% of relative intensity are in bold.
