# Supplementary material for: Characterization of the Mycobacterial Acyl-CoA Carboxylase Holo Complexes Reveals Their Functional Expansion into Amino Acid Catabolism
Source: PLoS Pathog. 2015 Feb 19;11(2):e1004623. doi: 10.1371/journal.ppat.1004623 (PMC4347857; doi:10.1371/journal.ppat.1004623)
Supplement: S4 Table — Restriction sites used for cloning are presented in bold face. CTTAAG = AflII, CCTAGG = AvrII, AAGCTT = HindIII, ACTAGT = SpeI, TCTAGA = XbaI, GGTACC = KpnI, CCATGG = NcoI. CCATGG = PstI, Up and Down signify the fragments homologous to the upstream and downstream region of the particular gene(s) that needed to be deleted. (DOC) [file ppat.1004623.s004.doc]

| **Primer** | **DNA sequence** | **Location 5’ end** | **Relative to** | **To create construct** |
| --- | --- | --- | --- | --- |
| Msmeg5492-F1 | GCGC**CTTAAG**TGGTCATCTGCGAGGAGATGCAC | -938 | *accD2* | pEN43-Up |
| Msmeg5492-R1 | GCGC**CCTAGG**CAGGGCAGTCATGACTGGAACCCC | +12 | *accD2* | pEN43-Up |
| Msmeg4717-F1(500) | GCGC**GGTACC**GTCAGCACCGACACCCAGATCTC | -515 | *accD1* | pEN30-Up |
| Msmeg4717-R1 | GCGC**TCTAGA**CAAGGCGTCTCCATCTGAGTTAATG | +3 | *accD1* | pEN30-Up |
| Msmeg4716-F2 | GCGC**AAGCTT**GCCCGTATCACCGCCCACACCC | +2082 | *accA1* | pEN30-Down |
| Msmeg4716-R2(500) | GCGC**ACTAGT**CGAGTTGGTGATGAACTGCTTGGAC | +2612 | *accA1* | pEN30-Down |
| Msmeg5493-dw-fwd | GCCC**AAGCTT**ACGATGAGCGGTTTCGTCGAGACC | +1964 | *accA2* | pEN43-Down |
| Msmeg5493-dw-rev | GCCC**ACTAGT**ACGGCCTGCGCCTGGTCGATGC | +2440 | *accA2* | pEN43-Down |
| MTE01 | CCGT**CCATGG**ACGTGACCACCCCGTCGATAGC | +1 | *accD1* | pMyNT D1A1 |
| MTE02 | GCG**AAGCTT**CTAGTCCTTGATCCTCGCC | +3559 | *accD1* | pMyNT D1A1 |
| MTE03 | CCGT**GGTACC**GTGCTGCAATCCACACTGG | +1 | *accD2* | pMyNT D2A2 |
| MTE04 | GCG**AAGCTT**TCACGGTGAATCTCCTTCTGC | +3599 | *accD2* | pMyNT D2A2 |
