# Supplementary material for: Characterization of the Mycobacterial Acyl-CoA Carboxylase Holo Complexes Reveals Their Functional Expansion into Amino Acid Catabolism
Source: PLoS Pathog. 2015 Feb 19;11(2):e1004623. doi: 10.1371/journal.ppat.1004623 (PMC4347857; doi:10.1371/journal.ppat.1004623)
Supplement: S5 Table — (DOC) [file ppat.1004623.s005.doc]

| **Plasmid/construct** | **Description** | **Reference** |
| --- | --- | --- |
| pJV53 | Che9c recombination proteins under control of the acetamidase promoter in pLAM12. | [35] |
| pYUB854 | Hyg^R^ cassette flanked by γδ–res sites and 2 MCSs. | [36] |
| pGH542 | Expressing an γδ resolvase and tetracycline resistant. | [37] |
| pEN30 | pYUB854 with a fragment harboring the upstream region of *accD1* inserted upstream of the Hyg^R^ cassette and a fragment of the downstream region of *accA1* inserted downstream of the Hyg^R^ cassette. | This paper |
| pEN43 | Idem as pEN29 but for *accD2-accA2.* | This paper |
| pMyNT | Mycobacterial expression vector, possessing a N-terminal His-tag. | A. Geerlof (unpublished) |
| pMyNT D1A1 | *M. tuberculosis accD1-accA1* in pMyNT, AccD1 is N-terminally His-tagged. | This paper |
| pMyNT D2A2 | *M. tuberculosis accD2-accA2* in pMyNT, AccD2 is N-terminally His-tagged | This paper |
