# Supplementary figures and images for: Characterization of the Mycobacterial Acyl-CoA Carboxylase Holo Complexes Reveals Their Functional Expansion into Amino Acid Catabolism
Source: PLoS Pathog. 2015 Feb 19;11(2):e1004623. doi: 10.1371/journal.ppat.1004623 (PMC4347857; doi:10.1371/journal.ppat.1004623)

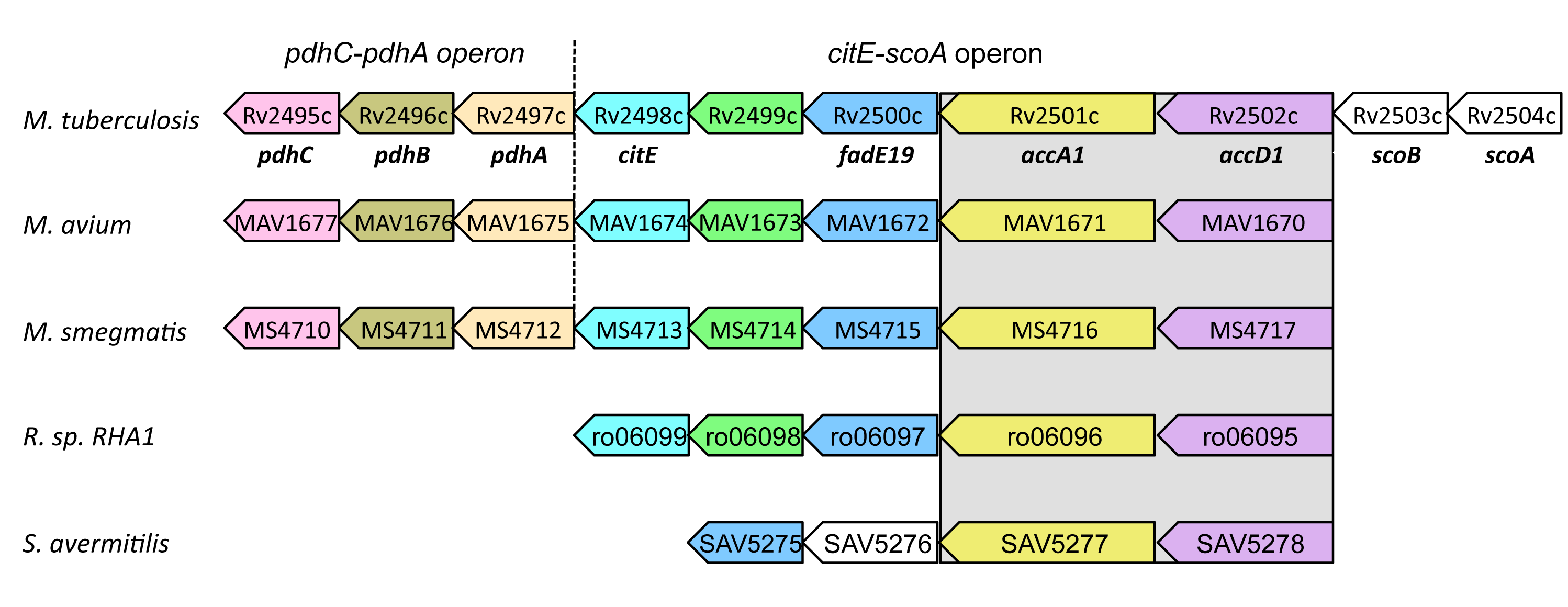

Supplement: S1 Fig — Structure of the M. tuberculosis citE-scoA operon (Rv2498c to Rv3504c) and the preceding pdhC-pdhA operon (Rv2495c to Rv25497c) and related operon structures in M. avium, M. smegmatis, R. sp. RHA1, and S. avermitilis. Homologous genes are shown in identical colors and are annotated with their standard gene identifiers. In addition, for all those M. tuberculosis genes with predicted or known function, additional functional gene codes are also presented. The genes Rv2591c (accD1) and Rv2502c (accA1) from M. tuberculosis, the subject of this contribution, and homologous genes from other shown organisms are boxed. (TIF) [file ppat.1004623.s006.tif]

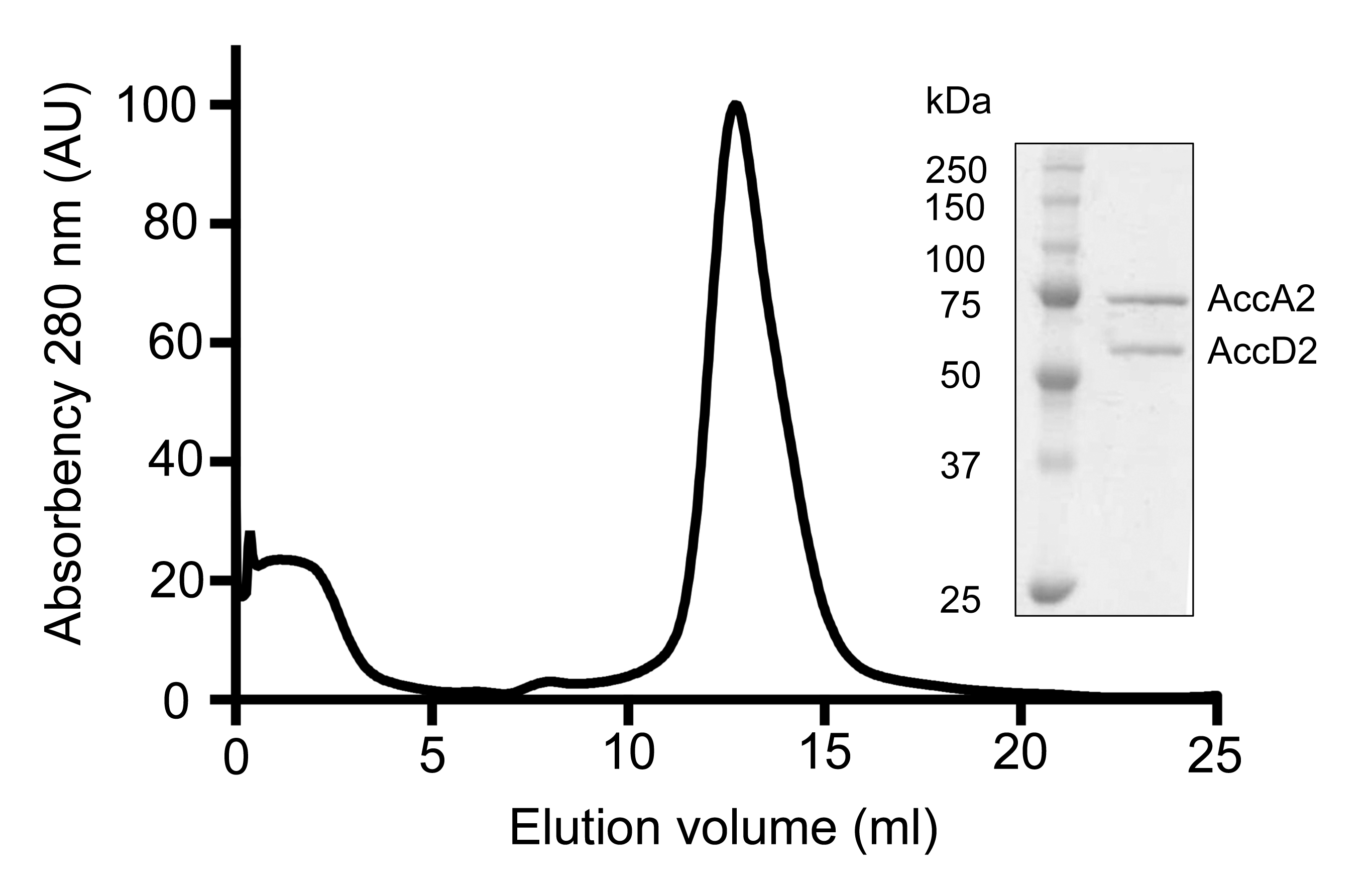

Supplement: S2 Fig — (TIF) [file ppat.1004623.s007.tif]

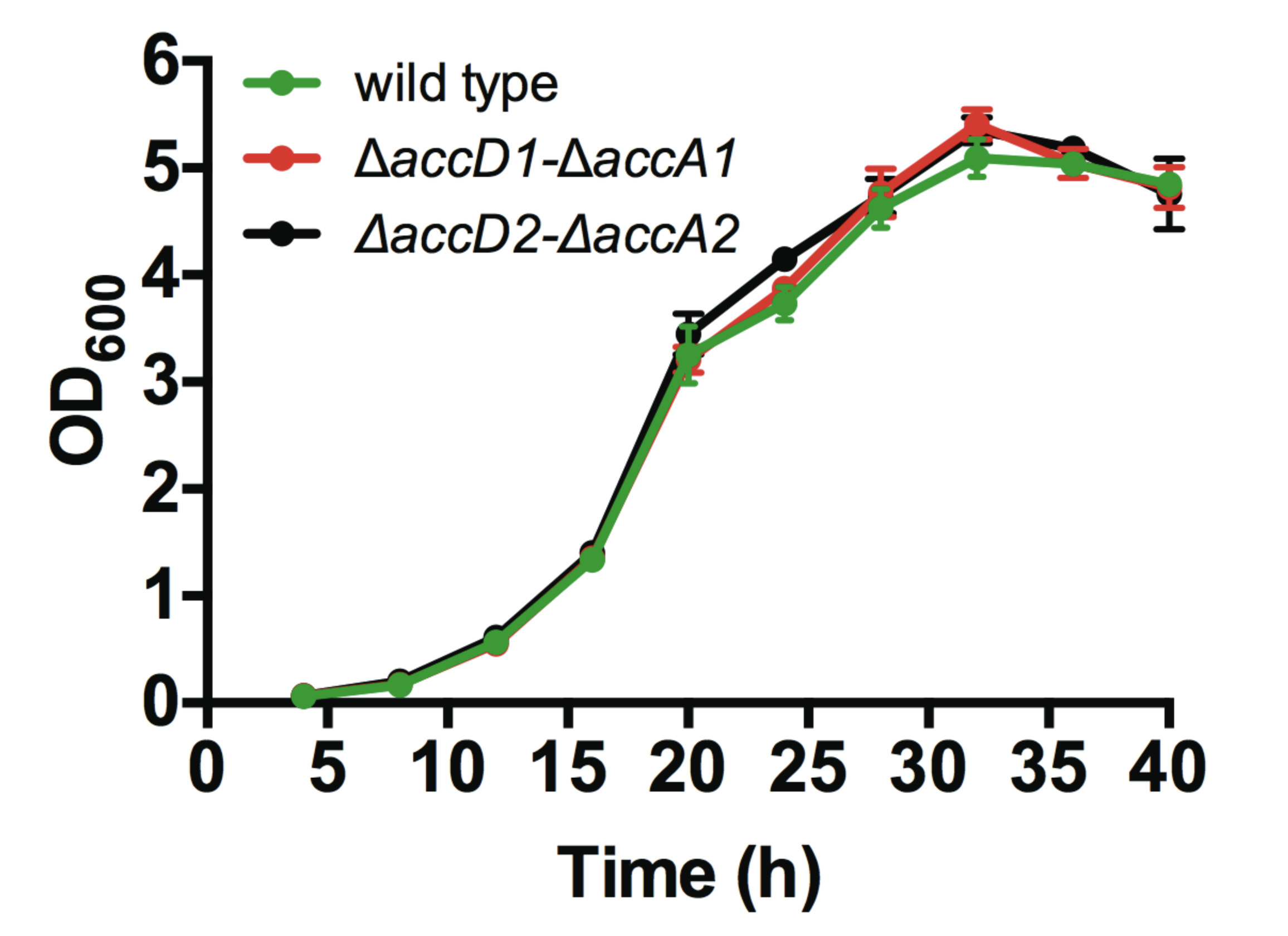

Supplement: S3 Fig — Batch cultures of each strain were grown in triplicate at 37°C and the cell density monitored by measuring the optical density of the culture at 600nm every 4 hours. The wt is in green, ΔaccD1-ΔaccA1 is in red, ΔaccD2-ΔaccA2 is in black. (TIF) [file ppat.1004623.s008.tif]

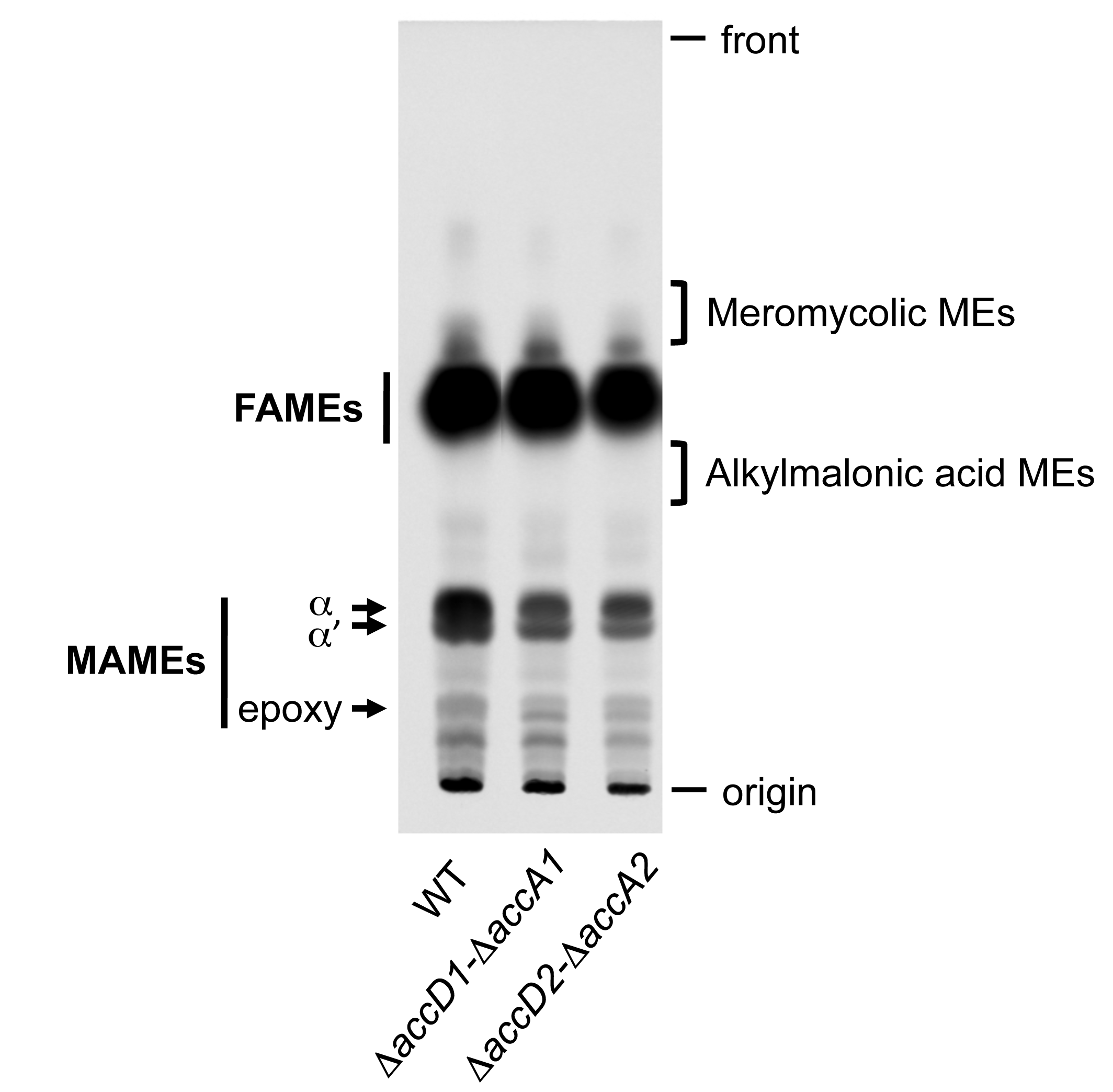

Supplement: S4 Fig — TLC analysis of labeled lipids (FAMEs and MAMEs) of wt and the knockouts strains after incubation with [1–14C]acetic acid. FAMEs: fatty acid methyl esters, MAMEs: mycolic acid methyl esters (with the three types of M. smegmatis mycolic acids alpha, alpha’ and epoxy). The expected migration of meromycolic acid- and alkylmalonic acid-methyl esters (ME) is indicated on the TLC. TLC was developed with dichloromethane and visualization performed by phosphorImager. (TIF) [file ppat.1004623.s009.tif]

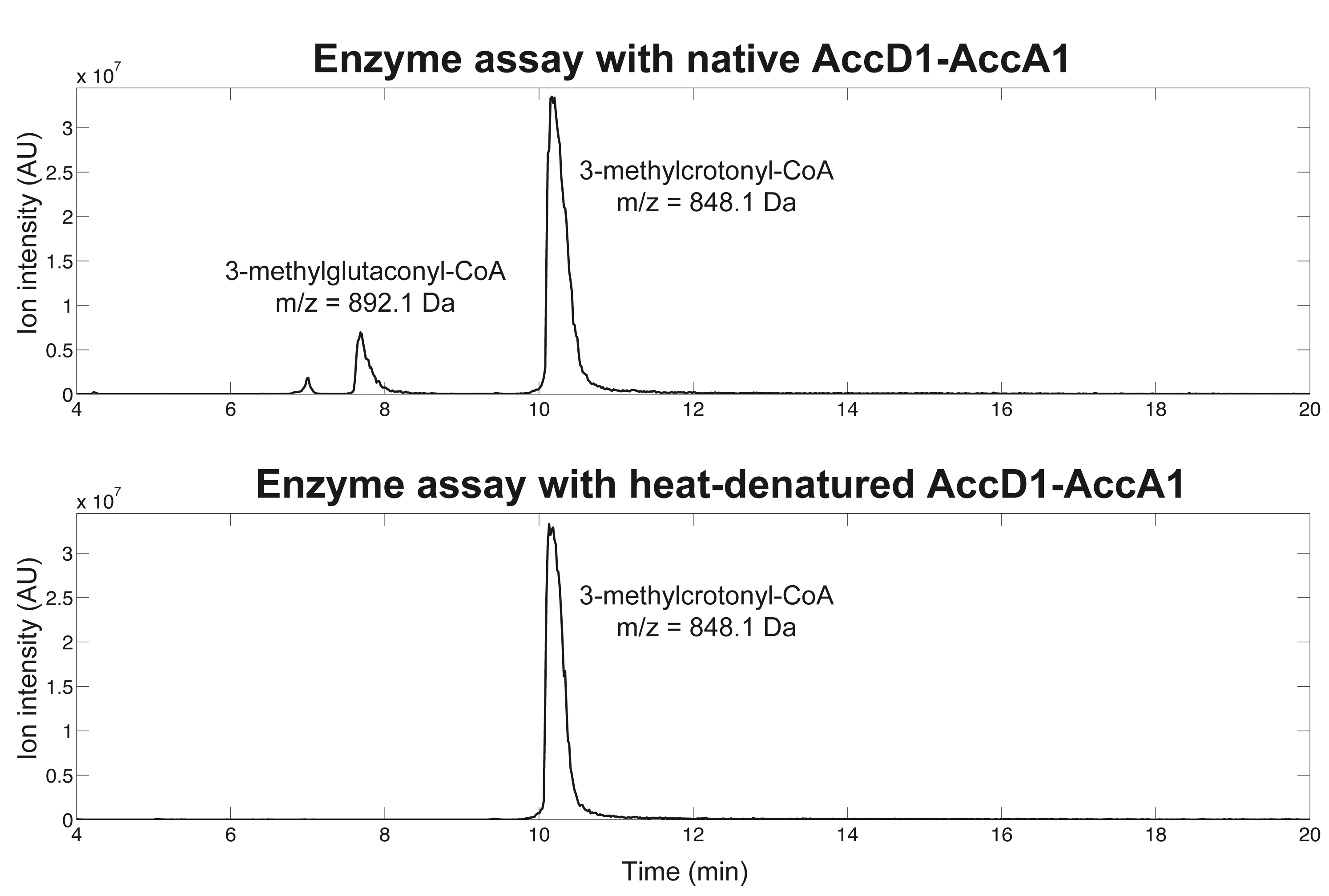

Supplement: S5 Fig — LC-MS/MS analysis of CoA thioester in samples containing putative substrate, 3-methylcrotonyl-CoA, and the enzyme AccD1-AccA1 as determined under the assay conditions described under Materials and Methods. After 30 min of incubation the assay mixture was snap-frozen and the CoA thioesters were analyzed by LC-MS/MS as described. Besides the substrate peak the production of an additional CoA thioester was detected in the sample containing the native enzyme (upper panel) but it was absent in the sample containing heat-denatured (10 min at 95°C) enzyme (lower panel). This CoA thioester corresponds to methylglutaconyl-CoA with an m/z of 892 Da of its deprotonated form. The mass difference of 44 Da between 3-methylcrotonyl-CoA and methylglutaconyl-CoA reflects the carboxylation reaction. (TIF) [file ppat.1004623.s010.tif]

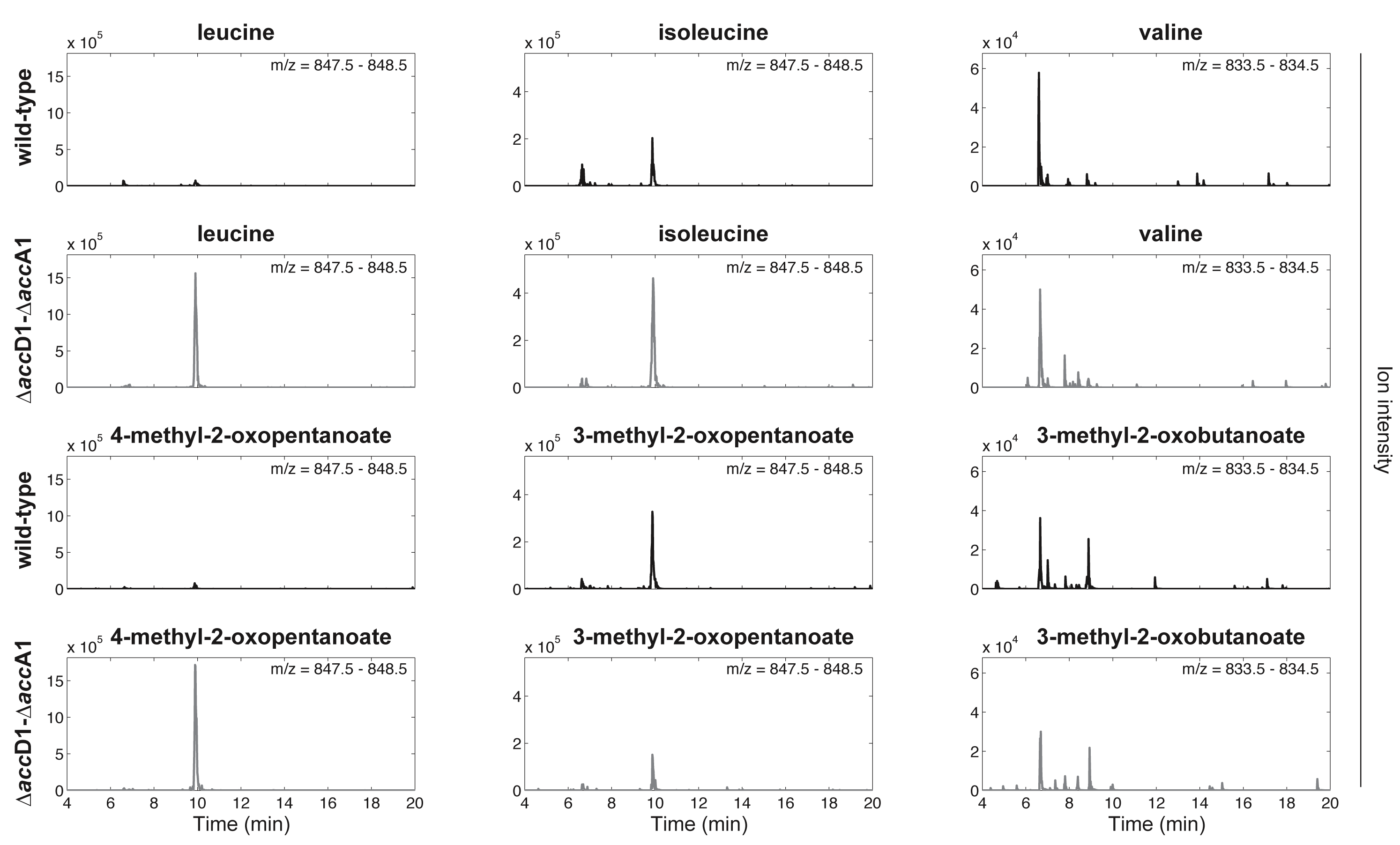

Supplement: S6 Fig — Nutritional shifts from cultures grown on glycine as sole carbon source to a culture medium containing leucine, 4-methyl-2-oxopentanoate, isoleucine, 3-methyl-2-oxopentanoate, valine, or 3-methyl-2-oxobutanoate as sole carbon source. One hour after the nutrient shift, intracellular metabolites were extracted and the CoA thioesters were analyzed by LC-MS/MS as described in the manuscript. The shift to either leucine or 4-methyl-2-oxopentanoate led to a striking accumulation of 3-methylcrotonyl-CoA in the ΔaccD1-ΔaccA1 strain. However, the shift to either valine or 3-methyl-2-oxobutanoate did not lead to the accumulation of methylacrylyl-CoA in the same strain, as no peak with an m/z of 834 Da in negative mode was significantly different in the ΔaccD1-ΔaccA1 strain compared to the wt strain. The shift to either isoleucine or 3-methyl-2-oxopentanoate did not lead to an accumulation of 2-methylcrotonyl-CoA with an m/z of 848 Da in the ΔaccD1-ΔaccA1 strain, as one would expect if AccD1-AccA1 were involved in the degradation of isoleucine. In conclusion, these results demonstrate that the principle role of AccD1-AccA1 in vivo is the carboxylation of 3-methylcrotonyl-CoA to methylglutaconyl-CoA as part of the leucine degradation pathway. (TIF) [file ppat.1004623.s011.tif]

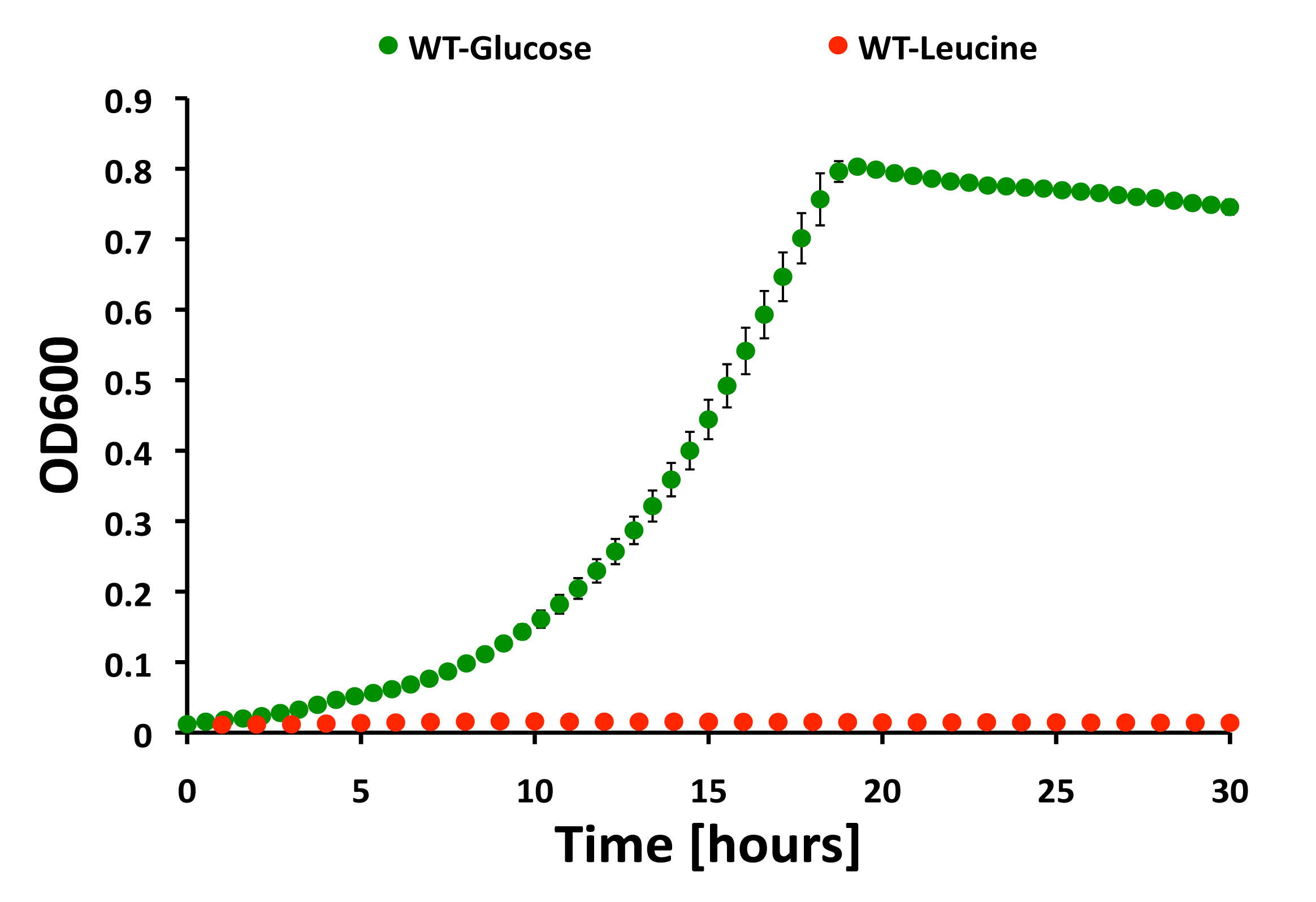

Supplement: S7 Fig — Average values for the culture density were calculated from four replicates. (TIF) [file ppat.1004623.s012.tif]

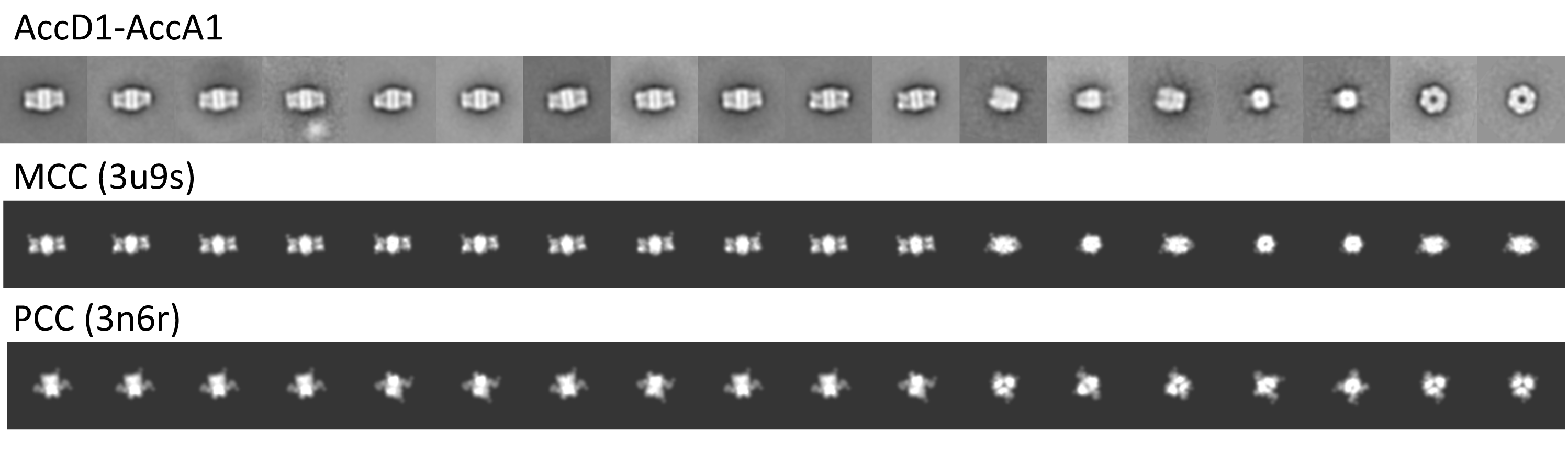

Supplement: S8 Fig — Simulated projections were generated from random orientations with five degree Euler intervals. Projections with highest correlation to selected AccA1-AccD1 class averages are shown in their corresponding orientation. The data demonstrate that the overall conformation of AccD1-AccA1 resembles the MCC complex and is substantially different from that of PCC. (TIF) [file ppat.1004623.s013.tif]

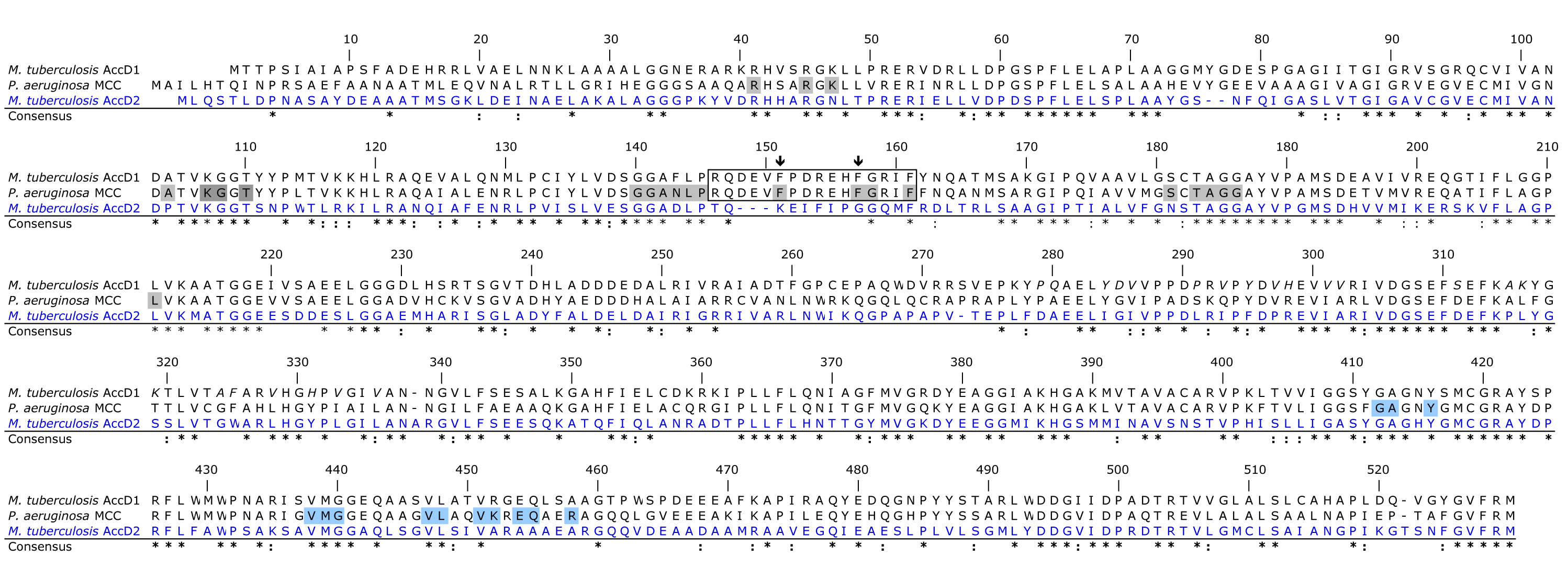

Supplement: S9 Fig — AccD1 from M. tuberculosis (O06165_MYCTU), MCC from P. aeruginosa (Q9I297_PSEAE), and AccD2 from M. tuberculosis (0T826_MYCTU). Residue numbers refer to the M. tuberculosis AccD1 sequence. The M. tuberculosis AccD2 sequence, which is likely to have different substrate specificity (for details, see text), is shown in blue. In the consensus line, invariant and conserved residues positions are marked by “*” and “:”, respectively. Residues that are involved in binding to 3-methylcrotonyl-CoA within the CT dimer interface of the substrate model of the MCC holo complex P. aeruginosa [17] are highlighted in grey and cyan, matching the color scheme of Fig. 7. The sequence motif “RQDEVFPDREHFGRIF” (residues 146–161 of the M. tuberculosis AccD1 sequence), which is invariant in M. tuberculosis AccD1 and P. aeruginosa MCC but divergent in M. tuberculosis AccD2, is boxed (cf. Fig. 7). Phe151, Phe157 and Phe161 (M. tuberculosis AccD1 numbering scheme) are critical in determining 3-methylcrotonyl-CoA binding specificity and are indicated by arrows. (TIF) [file ppat.1004623.s014.tif]

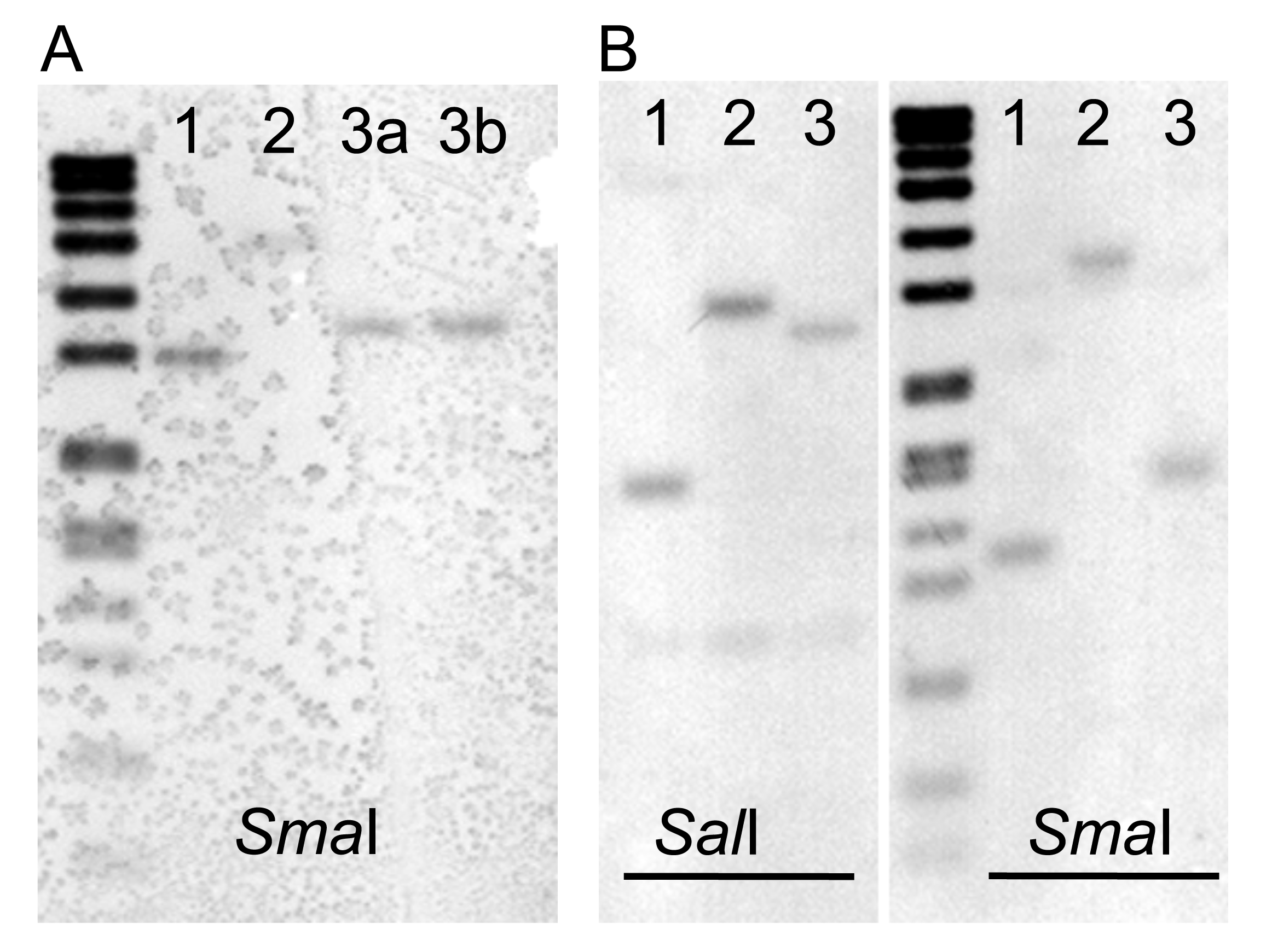

Supplement: S10 Fig — (A) Analysis of ΔaccD1-ΔaccA1 and (B) ΔaccD2-ΔaccA2, performed with genomic DNA of M. smegmatis mc2155 carrying pJV53 (lane 1), correct hygromycin-resistant mutants (lane 2) and correct unmarked deletion mutants (lane 3; a/b when more than one). The HindIII-SpeI digested fragment of pEN30 was used as a probe for ΔaccD1-ΔaccA1 and the AflII-AvrII of pEN43 for ΔaccD2-ΔaccA2. The genomic DNA was digested with restriction endonucleases shown in the figure. (M) DNA marker VII, digoxigenin labeled (Roche). (TIF) [file ppat.1004623.s015.tif]
